# Protocols and characterization data for 2D, 3D, and slice-based tumor models from the PREDECT project

**DOI:** 10.1038/sdata.2017.170

**Published:** 2017-11-21

**Authors:** Ronald de Hoogt, Marta F. Estrada, Suzana Vidic, Emma J. Davies, Annika Osswald, Michael Barbier, Vítor E. Santo, Kjersti Gjerde, Hanneke J. A. A. van Zoggel, Sami Blom, Meng Dong, Katja Närhi, Erwin Boghaert, Catarina Brito, Yolanda Chong, Wolfgang Sommergruber, Heiko van der Kuip, Wytske M. van Weerden, Emmy W. Verschuren, John Hickman, Ralph Graeser

**Affiliations:** 1 Janssen Pharmaceutica N.V., Turnhoutseweg 30, Beerse 2340, Belgium; 2 iBET, Instituto de Biologia Experimental e Tecnológica, Apartado 12, Oeiras 2780-901, Portugal; 3 Instituto de Tecnologia Química e Biológica António Xavier, Universidade Nova de Lisboa, Av. da República, Oeiras 2780-157, Portugal; 4 Institute of Biochemistry, Faculty of Medicine, University of Ljubljana, Ljubljana SI-1000, Slovenia; 5 Oncology IMED, Bioscience, AstraZeneca, 1 Francis Crick Avenue, Cambridge CB2 0AA, UK; 6 Boehringer Ingelheim RCV, GmbH & Co. KG, Dr. Boehringer-Gasse 5-11, Vienna 1120, Austria; 7 Institute of Medical Genetics, Medical University of Vienna, Waehringerstrasse 10, Vienna A-1090, Austria; 8 University of Antwerp, Department of Veterinary Sciences, Laboratory of Cell Biology & Histology, Universiteitsplein 1, Wilrijk 2610, Belgium; 9 ERASMUS MC, Wytemaweg 80, Rotterdam 3015 CN, The Netherlands; 10 Institute for Molecular Medicine Finland (FIMM), Tukholmankatu 8, University of Helsinki, 00014, Finland; 11 Dr Margarete Fischer-Bosch Institute of Clinical Pharmacology and University of Tuebingen, Auerbachstrasse 112, Stuttgart 70376, Germany; 12 AbbVie, 1 North Waukegan Road, North Chicago, IL 60064-6098, USA; 13 Institut de Recherches Servier c/o 126 boulevard Pereire, Paris 75017, France; 14 Boehringer Ingelheim Pharma GmbH & Co. KG, Birkendorfer Str. 65, Biberach an der Riß 88400, Germany

**Keywords:** Tumour heterogeneity, Target identification, Target validation, Cell growth, Cellular imaging

## Abstract

Two-dimensional (2D) culture of cancer cells *in vitro* does not recapitulate the three-dimensional (3D) architecture, heterogeneity and complexity of human tumors. More representative models are required that better reflect key aspects of tumor biology. These are essential studies of cancer biology and immunology as well as for target validation and drug discovery. The Innovative Medicines Initiative (IMI) consortium PREDECT (www.predect.eu) characterized *in vitro* models of three solid tumor types with the goal to capture elements of tumor complexity and heterogeneity. 2D culture and 3D mono- and stromal co-cultures of increasing complexity, and precision-cut tumor slice models were established. Robust protocols for the generation of these platforms are described. Tissue microarrays were prepared from all the models, permitting immunohistochemical analysis of individual cells, capturing heterogeneity. 3D cultures were also characterized using image analysis. Detailed step-by-step protocols, exemplary datasets from the 2D, 3D, and slice models, and refined analytical methods were established and are presented.

## Background & Summary

Carcinomas, also known as solid tumors, have a complex microenvironment, are cellularly heterogeneous, and have a three-dimensional (3D) architecture^1^. Ideally, laboratory models of these cancers should attempt to capture key aspects of this complexity and architecture. Despite the wealth of data generated, and strong recommendations to upgrade cell culture models from those growing in two dimensions (2D) (e.g., cellular monolayers on plastic substrates) to 3D models^2^, few of these more complex 3D model systems have been used to investigate cancer cell biology *in vitro*. Neither have they been routinely incorporated into the drug screening cascade alongside standard 2D models despite consistent evidence suggesting that preclinical models largely fail to predict drug efficacy^3^. Reproducibility, cost, and limited throughput are some of the issues precluding their routine use. A detailed characterization and cross-comparison of some more complex models with 2D models is pertinent. The public-private PREDECT consortium (www.predect.eu) set out to characterize an array of *in vitro* models for oncology research, attempting to construct models better able to capture the complexity of solid cancers^4^. Models were generated from a range of breast, prostate, and lung cancer cell lines as well as from patient-derived xenograft (PDX) and a genetically engineered mouse model (GEMM). Starting from conventional 2D monocultures, the complexity of the models was increased stepwise to include stromal cells in 2D co-cultures, and then in 3D cultures. The latter cultures were generated as free-floating spheroids (‘floaters’), microencapsulated into inert hydrogels (alginate) and grown in stirred-tank bioreactors (‘alginate-BR’), or embedded in extracellular matrix (ECM), all in the presence or absence of stromal cells^5–8^. Cell growth of the 2D/3D models, as well as their response to standard of care (SOC) drugs or chemotherapy were monitored by measurement of fluorescence. At stationary growth phase, (co-)cultures were analyzed in more depth by fluorescence imaging of *in situ* fixed cultures, as well as immunohistochemistry (IHC) on paraffin embedded samples processed into tissue microarrays (TMAs).

Precision-cut tissues slices derived from a GEMM or from PDX xenografts were also generated. The slices capture the native tumor microenvironment and any tumor heterogeneity that may exist and, as with the 2D and 3D models, were preserved as TMAs^9^.

The aim of this paper is to provide detailed descriptions of the protocols developed within the PREDECT consortium, methods to monitor culture viability status and to follow treatment responses. Moreover, raw data examples from PREDECT’s 2D/3D cell culture characterizations are provided for guidance. This guidance should allow other research groups to repeat and extend the data generated by the PREDECT consortium.

## Methods

**Figure 1:**
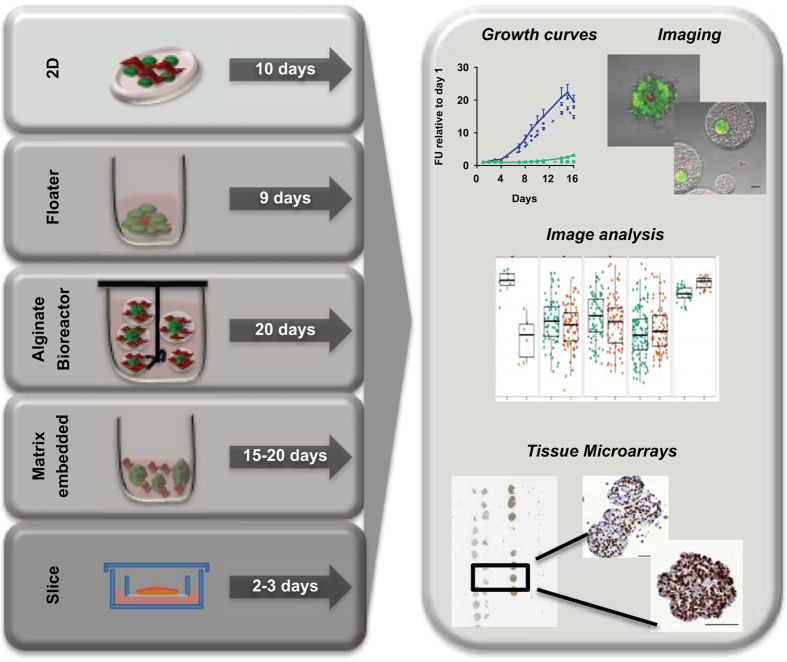
Models covered in this manuscript. A graphical representation of the cell culture platforms and their duration, as well as analyzes for which protocols and data are provided. Modified from ref. 5.

The methods section contains step-by-step protocols of the methods established and validated by the PREDECT consortium, starting with cultivation protocols and ending with analytical methods. An overview is presented in Fig. 1. These methods are expanded versions of descriptions in published work^5,9^.

### Tissue culture protocols

Cell lines used in the 2D and 3D experiments were transduced with genetic constructs driving expression of fluorescent proteins, in order to allow monitoring of the cells during cultivation. Since no common protocol to generate labeled cell lines was generated, but a variety of working protocols exist (see also^5^), this part of the procedure will not be described in detail here.

#### 2D cell culture.

2D cell cultures should be plated in black 96-well clear-bottom microplates (e.g., Greiner Bio One #655-088). All the different plates used in our study are listed below in Table 1. When performing experiments with several 96-well plates, drawing the layout of each plate on the lids simplifies and speeds up the pipetting process. The outer wells should not be used due to the evaporation edge effect during long term culturing.

**Step 1:** Prepare fresh cell culture medium without phenol red prior to each experiment.

**Step 2:** Trypsinize and collect tumor cells and fibroblasts in 50 ml tubes, centrifuge 3 min at 450×g. Resuspend cell pellets in 1–5 ml medium depending on the cell lines used.

*If experiments are conducted at a lower serum concentration than during regular culture, resuspend cell pellets in serum-free medium, centrifuge once more and resuspend in medium containing the desired serum concentration.

Determine the concentration for each cell line and prepare adequate dilutions for monocultures and co-cultures in medium, calculating 200 μl per well.

*Examples for cell numbers and ratios are presented in Table 2. The cell number and ratio for every new cell line/combination will have to be optimized. For tumor cell numbers, extremes of 5-times higher or lower than suggested in Table 2 may be tested, for ratios, a useful range is between 10:1 and 1:10.

**Step 3:** Seed the desired number of cells per well in 200 μl medium with all supplements required. Add 200 μl medium only to each control well.

*Pipetting with a multichannel pipet from a reservoir is an easy and fast approach to fill the plates.

**Step 4:** Fill the outer and additional empty wells with 200 μl of PBS before placing the plates in a humidified incubator at 37 °C and 5% CO_2_. Do not refresh the medium during cultivation as this may cause cells to detach and disturb the fluorescence growth measurements.

**Fluorescence measurements**

**Step 5:** Measure the baseline fluorescence values after overnight incubation with a plate reader. See Table 3 for plate readers used in this study.

*Excitation and emission settings depend on the construct the cells have been transduced with, e.g., 540/587 nm for RFP and 488/525 nm for GFP. See Table 3 for additional settings.

Repeat measurements every 2–3 days for growth curve construction until the cultures reach stationary phase.

**Drug treatment**

**Step 6:** Seed cells in 100 μl medium per well and add after overnight incubation 100 μl of 2X compound concentration to each well.

*Desired compound concentrations should be prepared in DMSO, unperturbed controls should receive medium with the same DMSO concentration. Since cell lines may react differently to the concentration of DMSO, maximally tolerated DMSO concentrations should be determined before starting perturbation experiments.

Measure the baseline fluorescence values and repeat every 2–3 days. Do not refresh the medium, unless absolutely required for cell growth.

#### 3D floater cultures.

3D floater cultures may be cultivated in 384 well ultra-low attachment (ULA; Corning #3830) or agarose-coated plates (Greiner Bio-One #781090). Supplementary Figure S1 illustrates a suggestion of a layout to seed tumor cells in mono- and two different co-cultures with stromal cells in a single 384 well plate.

**Step 1:** Prepare fresh cell culture medium without phenol red prior to each experiment.

When working with agarose-coated plates, coat each well with 10 μl 1.5% agarose in medium, seal the plates after 2 h with an adhesive cover and store overnight at 4 °C.

**Step 2:** Trypsinize and collect tumor cells and fibroblasts in 50 ml tubes, centrifuge for 3 min at 450×g. Resuspend cell pellets in 1–5 ml medium depending on the cell lines used.

*If experiments are conducted at a lower serum concentration than during regular culture, resuspend cell pellets in serum-free medium, centrifuge once more and resuspend in medium containing the desired serum concentration.

**Step 3:** Determine the concentration for each cell line and prepare adequate dilutions for monocultures and co-cultures in medium, calculating 50 μl per well for an ULA plate or 40 μl for an agarose-coated plate. Pre-warm agarose-coated plates to RT before seeding the cells.

*Examples for cell numbers and ratios are presented in Table 2. The cell number and ratio for every new cell line/combination will have to be optimized. For tumor cell numbers, extremes of 5-times higher or lower than suggested in Table 2 may be tested, for ratios, a useful range is between 10:1 and 1:10.

Mix the suspension well, and transfer 50 μl to each well of an ULA plate or 40 μl to an agarose-coated plate. Fill the control wells with 50 μl per 40 μl medium and outer wells with PBS.

**Step 4:** Centrifuge the ULA plate at 380×g (agarose-coated at 170×g) for 1 min at RT. Incubate the plate in a humidified incubator at 37 °C. Carefully refresh half of the medium twice a week. This can be performed by a washer or manually with a multichannel pipette.

*When refreshing the plates manually take great care not to lose the spheroids in the process. One option to prevent this is pipetting the old medium to a second plate and then measure the fluorescence in the second plate in order to identify wells where floaters accidentally have been removed.

**Fluorescence measurements**

**Step 5:** Measure the baseline fluorescence values after overnight incubation with a plate reader. See Table 3 for plate readers used in this study.

*Excitation and emission settings depend on the construct the cells have been transduced with, e.g., 540/587 nm for RFP and 488/525 nm for GFP. See Table 3 for additional settings.

Repeat measurements every 2–3 days for growth curve construction until the cultures reach stationary phase.

**Drug Treatment**

**Step 6:** When tumor cells enter exponential growth, add 25 μl medium including the compound at 3X concentration (should result in 1X concentration in 75 μl). Refresh half of the medium with 1X compound concentration twice a week.

#### 3D alginate-embedded stirred-tank bioreactor cultures (alginate-BR).

This protocol describes the set-up for one 125 ml spinner vessel with straight blade paddle impeller (Corning Life Sciences; BR).

**Step 1:** Prepare fresh cell culture medium without phenol red prior to each experiment.

**Step 2:** Collect the tumor cells as described in the 2D cell culture section and determine cell concentration. Inoculate single cell suspension (0.2×10^6^ cell per ml) in the 125 ml BR and induce cell aggregation by stirring the culture at 80 rpm, for 24 h.

*Stirring and aggregation time need to be adjusted for each cell line. The goal is to induce compacted spheroids with a diameter of 100 to 300 μm within 4 days of culture, while limiting clumping and fusion^7^.

**Step 3:** Prepare the microencapsulation solutions: 0.9% NaCl (w/v) and 100 mM CaCl_2_/10 mM HEPES (pH 7.4) or 20 mM BaCl_2_ (for full buffer composition, see step 4). Prepare the Ultrapure Ca^2+^ MVG alginate (PRONOVA UP MVG, #4200106 NovaMatrix, Pronova Biomedical, Oslo, Norway) by dissolving the alginate powder in NaCl 0.9% (w/v) solution to a final concentration of 1.1% (w/v). Shake the alginate mixture overnight at 20 rpm.

**Step 4:** Collect tumor spheroids from the BR, centrifuge at 50×g, 4 min, RT. Remove the supernatant, wash with PBS without calcium and magnesium, and centrifuge the spheroid suspension as before. Disperse tumor spheroids corresponding to 25×10^6^ cells in 3 ml of 1.1% (w/v) Ultrapure Ca^2+^ MVG alginate. For co-cultures, add stromal cells at this point, in a 1:1 proportion, to attain 50×10^6^ total cells.

*The low tumor:stroma cell ratio (1:1) in the inert alginate hydrogels was selected since no overgrowth by fibroblasts has been observed in any of the tumor:stroma combinations tested so far, and higher tumor:stroma cell ratios appeared to fail to appropriately stimulate tumor cells.

Microencapsulate using an electrostatic bead generator (Nisco VarV1, Zurich, Switzerland), and a nozzle of 250 μm inner diameter, to obtain beads of a diameter of approximately 500 μm.

Alginate beads are cross-linked in either a 100 mM CaCl_2_/10 mM HEPES (pH 7.4) solution for 10 min or a 20 mM BaCl_2_ solution, adjusted to 290–300 mOsm using NaCl, buffered at pH 7.4 with 5 mM histidine, for 10 min.

*Calcium crosslinking has the advantage of being non-toxic. Also, it is easier to chelate and thus recover embedded cells. However, calcium cannot be used with all culture media (e.g., in RPMI1640, calcium-crosslinked capsules are chelated only after few days of culture).

*Barium crosslinking can be used with all culture media and provides stiffer capsules. However, it is harder to chelate and thus more difficult to recover embedded cells. Also, toxicity is higher in case of excessive crosslinking.

The microencapsulated cultures are then washed three times in a 0.9% (w/v) NaCl solution and finally equilibrated in culture medium before being transferred to the BR. Keep the microencapsulated mono- and co-cultures in a humidified incubator, at 37 °C and 5% CO_2_, for 20 days, with 50% medium exchange every 3–4 days.

**Fluorescence measurements**

**Step 5:** After overnight incubation, remove a sample from the bioreactor to measure the baseline fluorescence values with a plate reader. See Table 3 for plate readers used in this study.

*Excitation and emission settings depend on the construct the cells have been transduced with, e.g., 540/587 nm for RFP (dTomato) and 488/525 nm for GFP. See Table 3 for additional settings.

Repeat measurements every 2–3 days for growth curve construction until the cultures reach stationary phase.

**Drug Treatment**

**Step 6:** In order to determine dose-response curves and IC_50_/IC_80_ values, transfer a sample of the alginate-BR cultures to 96-well plates when at exponential phase of growth.

*Using a small calibration curve, 10 capsules per well was determined to be in the middle of the linear phase of the curve, thus allowing for a quantification of both signal increase and decrease. In addition to an equal number of capsules, the baseline fluorescence signal was used to normalize the inter-well variability.

Add the test compound at different concentrations. Refresh 50% of the medium every 3–4 days of culture.

*It is recommended to determine dose-response curves using this strategy. Once the drug concentration of interest is known, the drug may be added directly to the stirred-tank culture.

#### 3D matrix-embedded cell culture.

This protocol is for approximately three 96-well plates with Matrigel, Matrigel-collagen (mixed), and collagen cultures. Remaining Matrigel can be stored at 4 °C but we recommend preparing fresh Matrigel for important experiments. 3D cell cultures should be plated in black 96-well clear-bottom microplates (see Table 1). In the present protocol FBS was used at a concentration of 2%, as we aimed to keep serum concentrations low to detect a possible crosstalk between tumor cells, stromal cells and extracellular matrix components. See Supplementary Fig. S2 for a suggested plate layout.

**Step 1:** Prepare fresh cell culture medium without phenol red prior to each experiment.

Leave Matrigel to thaw on ice, this can be done overnight if kept at 4 °C.

*Prepare Matrigel (Corning #356231 10.8 mgml^−1^) solution by diluting one 10 ml vial to 8 mgml^−1^ with 3060.2 μl 1X RPMI, 135 μl 100X Glutamax (2 mM), 34.8 μl 45% D-glucose (12 mM) and 270 μl FBS (2%). Keep on ice in a 50 ml tube. When working with Matrigel at a different initial concentration the dilution process will need to be adjusted to maintain the final concentration of the additives.

*Prepare 10 ml of a 3 mgml^−1^ collagen I (BD Bioscience # 354236 3.8 mgml^−1^) solution containing 1 ml 10x RPMI, 300 μl 1 M HEPES, 300 μl 7,5% sodium bicarbonate and 8 ml collagen in a 50 ml tube. Add 1 M NaOH until the pH is between 7.1–7.4, then add 200 μl FBS, top up to 10 ml with H_2_O, and mix gently. Keep the solution on ice.

Pre-coat the wells with 30 μl of relevant matrix diluted 1:1 with RPMI (final concentration: Matrigel 4 mgml^−1^ and collagen 1.5 mgml^−1^) and centrifuge the plates 1 min at 975×g to ensure an even and complete coating of the whole well bottom. For mixed matrix dilute Matrigel with collagen 1:1. Place the pre-coated plates in a humidified incubator at 37 °C for at least 30 min to allow solidification of the matrix before adding the cell suspension.

*This helps to prevent 2D growth of the cells on the bottom of the wells.

**Step 2:** Trypsinize and collect tumor cells and fibroblasts in 50 ml tubes, centrifuge 3 min at 450×g. Resuspend cell pellets in 1–5 ml medium depending on the cell lines used.

*If experiments are conducted at a lower serum concentration than during regular culture, resuspend cell pellets in serum-free medium, centrifuge once more and resuspend in medium containing the desired serum concentration.

**Step 3:** Determine the concentration for each cell line and prepare adequate dilutions for monocultures and co-cultures in medium, based upon 30 μl per well.

*Examples for cell numbers and ratios are presented in Table 2. The cell number and ratio for every new cell line/combination will have to be optimized. For tumor cell numbers, extremes of 5-times higher or lower than suggested in Table 2 may be tested, for ratios, a useful range is between 10:1 and 1:10.

Mix well before adding the same volume of relevant matrix. Mix gently with a multichannel pipette and transfer 60 μl to each well. Add 60 μl matrix only to control wells and place the plates in a humidified incubator at 37 °C for at least 90 min.

*It is recommended to put the assay plate on a heating block when preparing the cultures as this will speed up setting of the matrix, and prevent the cells from sinking to the bottom of the well.

*When preparing mixed matrix, prepare the cell suspension in collagen and add an equal volume of Matrigel. 10% extra-volume should be prepared due to the viscosity of the matrices.

**Step 4:** Add 90 μl of medium at room temperature (RT) on top of the matrix in each well. Fill the outer and additional empty wells with 200 μl of PBS before placing the plates back in the humidified incubator at 37 °C/5% CO_2_. Refresh the medium twice a week, preferably with the plates placed on a heating block to prevent temperature drops. Tilt the plates slightly when removing the medium to prevent damaging the 3D matrix.

**Fluorescence measurements**

**Step 5:** Measure the baseline fluorescence values after overnight incubation with a plate reader. See Table 3 for plate readers used in this study.

*Excitation and emission settings depend on the construct the cells have been transduced with, e.g., 540/587 nm for RFP and 488/525 nm for GFP. See Table 3 for additional settings.

Repeat measurements every 2–3 days for growth curve construction until the cultures reach stationary phase.

**Drug Treatment**

**Step 6:** When tumor cells enter exponential growth, remove the overlay medium and add a 2X compound concentration (to compensate for the matrix volume) in 90 μl medium. Refresh the medium with 1X compound concentration twice a week.

#### Tissue slice cultures from tumors grown in mice.

Clean all instruments with 70% EtOH before use.

**Step 1:** Prepare media plates (described in Tables 1/4), placing 1.3 ml of media into each well of a 6 well plate, or 0.5 ml into a 24 well plate. Then place an organotypic support in each well, and allow for the system to equilibrate

*6 and 24 well organotypic support versions were used (Millipore, PICM 0RG 50 & PICM 012 50).

**Step 2:** Cull mouse and clean skin with 70% EtOH, using sterile dissection kit dissect tumor and place immediately into ice cold Oncostore solution (OncoScience AG) or L-15 Leibovitz Medium (Lonza)

**Step 3:** Prepare the vibrating microtome for use, cleaning all reusable parts with 70% EtOH.

*A Leica VT1200S vibratome was used for all tissue slice experiments in this manuscript. Other devices are available, however, methods have to be adapted according to the manufacturer.

*Leica’s Vibrocheck device was used prior to each use to calibrate the vertical deflection, ensuring even cutting of the blade

**Step 4:** Embed tumor in low temperature melting agarose in the desired orientation

*Remove excess connective tissue and liquid

*This step depends on tumor size and shape. If big enough and evenly shaped, the tumor does not have to be embedded.

**Step 5:** Mount (embedded) tumors on the sample platform using cyanoacrylate glue (e.g., Rot1 Coll 1 (Carl Roth GmbH)).

*If using another vibrating microtome, place tumor in the cutting chamber.

Place sample platform with tissue in buffer tray, fill with cold PBS supplemented with penicillin (100 Uml^−1^; e.g., Gibco) and streptomycin (100 μgml^−1^; e.g., Gibco) to cover the tumor sample.

**Step 6:** Set slicing parameters and begin slicing.

*Settings need to be adjusted to the sample. The softer the tissue, the higher the amplitude and lower the speed setting that should be used.

*As a starting point, these settings may be used: speed: 0.2 mm/s, amplitude: 1 mm, slice thickness: 250 μm

Use a probe to aid complete severance of the slice from the tumor.

**Step 7:** Remove slices from the machine using a spatula and probe, being careful not to pierce the slices, and place them on organotypic supports.

*Avoid trapping air bubbles underneath the slice

*Ensure that filter supports are moistened with medium before use.

**Step 8:** Add a few drops of media on the air surface of the slice to ensure sample is moistened (see Table 4 for media used).

**Step 9:** Place culture plate with slices in incubator

*37 °C and 5% CO_2_ in a humidified atmosphere under low oxygen (3–5% oxygen) or atmospheric oxygen (21% oxygen)

**Step 10:** Remove 1/2 media every 24 h from the bottom of the well, adding 50 μl of media on top of the slice to keep it moistened.

### Analytical methods

#### Generation of growth curves for 2D/3D cell cultures

Tumor and stromal cell growth in live cultures was simultaneously monitored using their distinct fluorescent protein markers until stationary phase was reached. Here is a quick guide how to normalize the data, and establish corresponding growth curves. For tissue slice culture the need to cultivate tissue slices on filter support, and the thickness of the slices rendered it impractical to apply a similar strategy to follow tumor cell behavior in real-time.

**Step 1:** For both fluorescence spectra, calculate the means of the growth medium/matrix controls, which serve as a background control, and divide or subtract all wells within the plate by this value, as indicated below in formulas 1 and 2.

**Step 2:** Calculate the relative growth over time, and then normalize to the reading at day 1.

*Formula 1: (fluorescence_day X/Average fluorescence of Background day X)/(fluorescence day 1/Average fluorescence of Background day 1).

*Formula 2: (fluorescence day X- mean background fluorescence day X)/(fluorescence day 1- mean background fluorescence day 1).

*Templates to process raw fluorescence intensities to background corrected day 1 normalized values are provided, and also sample sets of raw data (see raw data EXCEL sheets). The templates are generic and could be used to process different matrixes, compound treatments and fluorescent wavelength readings.

**Step 3:** Import normalized values to Graphpad Prism (or similar) for visualization of the growth curves.

**Step 4:** Several statistical tests/procedures were used for an analysis of the growth curves.

*Test for normality using the Shapiro-Wilks test.

*Non-parametric analyzes were done with the Mann-Whitney-*U*-Test, parametric with the *t*-test. Multiple groups were compared using a one-way ANOVA using the Tukey post-hoc test. Significances are depicted as n.s.: not significant, *P<0.05, **P<0.01, ***P<0.001.

EXCEL files containing exemplary datasets for 2D and 3D models have been uploaded to Figshare (see data records section).

#### Cell proliferation staining of 2D and 3D matrix embedded cell cultures

Cells were stained with Click-iT EdU Alexa Fluor 647 HCS Assay (2 plate kit, Life Technologies C10356) and Hoechst 33342 (Invitrogen, H3570). Growth medium, cell density, cell type variations, and other factors may influence labeling. In initial experiments, it is recommended to test a range of EdU concentrations to determine the optimal concentration for your cell type and experimental conditions. A similar concentration to BrdU is a good starting concentration for EdU. Whilst BrdU labeling in combination with IHC analysis may be a workable way forward, the fluorescence-labeled EdU was not used on tissue slices.

**Step 1:** Prepare stock solutions according to the Click-iT EdU Alexa Fluor 647 HCS Assay manual.

**Step 2:** Prepare a 2X working solution of EdU (Component A) in medium from the 10 mM stock solution. Suggested starting concentration is 10 μM. Pre-warm the 20 μM EdU and add an equal volume of 20 μM EdU solution to medium.

*Use the same medium as in the 2D/3D experiments.

**Step 3:** Add 100 μl working solution to each well and incubate the cells for 2 h at 37 °C.

*This incubation time should be adjusted according to the proliferation rate of a given cell line.

**Step 4:** Gently remove the medium from the wells making sure not to damage the 3D matrix. Tilt the plate slightly and keep the tip of the pipette close to the wall of the well. This will prevent disturbing the 2D monolayer of cells. Slowly add 150 μl formaldehyde (4%) to the side of each well and incubate for 30 min at RT.

**Step 5:** Remove the fixative gently and wash the wells three times with 150 μl of PBS.

*The samples can now safely be stored at 2–6 °C.

**Step 6:** Remove the wash solution and add 150 μl of 0.1% Triton X-100 in PBS to permeabilize the cells. Incubate 30 min at RT.

**Step 7:** Gently remove the Triton X-100 solution from the wells and wash with 150 μl PBS with two quick washing steps followed by two long washing steps (2×15 min). Make sure to remove the wash solution completely from each well.

**Step 8:** Perform the EdU detection according to the Click-iT EdU Alexa Fluor 647 HCS Assay manual.

**Step 9:** Wash each well with 150 μl PBS.

Dilute Hoechst 33348 in PBS (1:1,000) and add 100 μl to each well. Incubate 30 min at RT wrapped in aluminum foil.

Wash three times with PBS for 10 min. Fill up the wells with PBS.

*At this point the samples can be safely stored at 4 °C for a few weeks until analysis.

Seal the plate with aluminum foil to prevent liquid evaporation and bleaching.

Examine the sample with a suitable microscope.

#### Image analysis of fluorescence-labeled 3D cell cultures

For the analysis of 3D cultures, a variety of techniques, both to acquire and analyze images, have been validated and further developed. Up to now, no image analysis for whole tissue slices has been established yet.

The approaches for the analysis of 3D cultures must be adapted to sample type and microscopes used. Sample type parameters to be considered are:

- Sample depth: This parameter primarily impacts 3D-embedded cultures. The maximum depth that can be acquired is typically ca. 1 mm but this is heavily dependent on the microscope settings (i.e., objectives used).
- Sample density: This takes into consideration the minimal distance between spheroids (and therefore nuclei). The density in 3D needs to be carefully adjusted to achieve sufficient objects per image (for statistical analyses), but light transmission from objects above and below should be taken into consideration. ‘Light pollution’ and shadowing effects from objects outside of the focal plane, will impact both the segmentation efficiency and the feature values extracted from the micrographs.
- Spheroid size: Larger spheroids (>100 μm) are subject to light attenuation due to light scattering inside the cell mass, therefore preventing proper imaging of the inner and the deeper parts of the spheroids.

**Figure 2:**
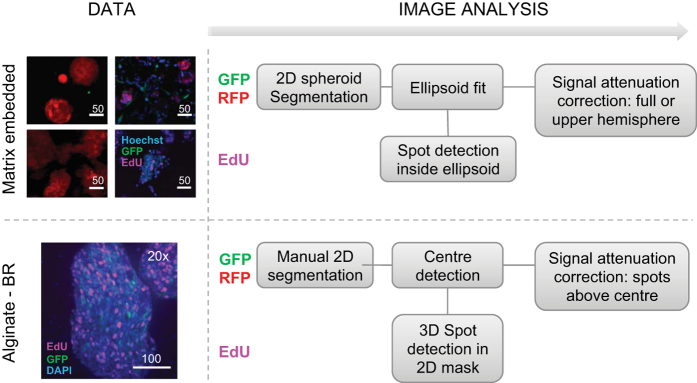
Image analysis workflow of 3D spheroid tumor cultures. Upper panel, matrix embedded cultures; lower panel as for alginate-BR cultures. RFP (red): Tumor cells; GFP (green): Stromal fibroblasts; DAPI (blue): Nuclei; EdU (pink): Proliferating nuclei.

Images of the spheroid cultures may be analyzed using software algorithms developed in MATLAB for this purpose^10^. The pipeline to analyze Alginate-BR and matrix embedded (co-) cultures is illustrated in Fig. 2. For each of the 3D culture methods an example workflow, images and analysis parameters are provided at Figshare, along with a description and an overview table.

#### 3D Floater cultures

Floater cultures contain a single, relatively large and almost spherical spheroid per well with area sizes of up to 0.5 mm^2^. Signal attenuation within these large spheroids cannot be addressed post-imaging, but solutions to improve light penetration proved unsuccessful in our hands^5^. A pragmatic alternative is to apply 2D projected spheroid size and shape analyzes. However, analysis of EdU positive cells, or full 3D analysis appear to be restricted to smaller spheroids for the time being.

2D size and shape analyzes are carried out in an automated fashion using the algorithm provided on (Data Citation 1) and entitled ‘MATLAB script for the image analysis of 3D image stacks of 3D multi-cellular spheroid cultures’.

**Step 1:** Place plate under microscope.

**Step 2:** Acquire wide-field images

*When spheroids are large and analysis of EdU positive cells is not feasible because of signal attenuation, wide-field microscope images are preferred over confocal images. Otherwise, when spheroids are small (below 200 micron), confocal image stacks should be taken, and analysis protocols should be followed as described for the alginate BR.

**Step 3:** Analysis: For 2D size and shape analysis the CellProfiler pipeline provided on (Data Citation 1) and entitled ‘Example CellProfiler pipeline for the image analysis of the breast(lung, prostate) cancer cell bioreactor cultures’ is used.

#### 3D alginate-BR cultures

These cultures generate spheroids with a maximal area size of 0.01–0.1 mm^2^, which, in terms of spheroid sizes, places them between matrix embedded and floater cultures. The shape of the spheroids is often irregular, especially when cells leak out of the alginate micro-capsules. The density of EdU-positive nuclei within these spheroids is relatively high.

**Figure 3:**
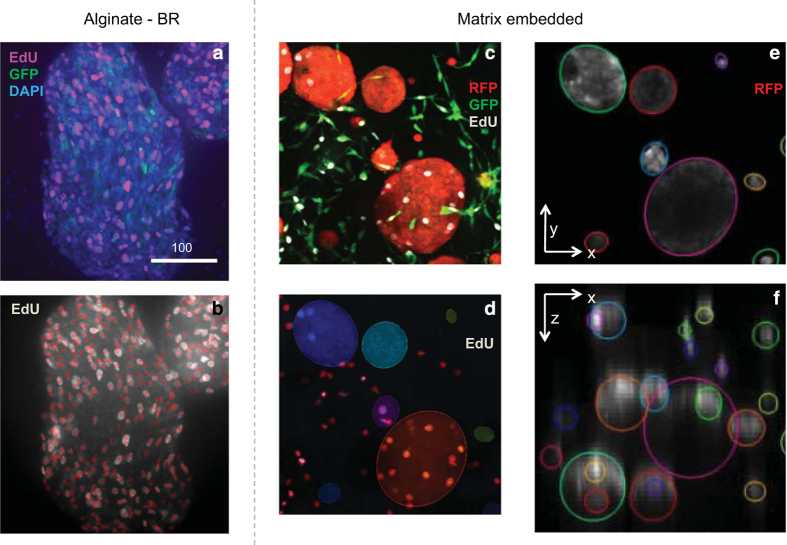
Examples for the image processing workflow. (**a**,**b**) A fluorescent spheroid from an alginate-BR culture with (**b**) the detection of EdU positive cell illustrated, where red circles indicate positive cells. (**c**) A small subset of an image-stack of a matrix (Matrigel, DMSO) embedded 3D spheroid culture, where in panel (**d**) the detection of EdU positive cells is shown as circles within spheroids (red circles outside the spheroid originate from proliferating fibroblasts). The spheroids themselves are approximated by ellipsoids and shown in color. In (**e**,**f**) the top and side view of the segmentation contours are shown to illustrate the ellipsoid approximation. Spheroids only visible in (**f**) but not in (**e**) are originating from (x,y)-coordinates outside the subset of the xy-plane. RFP (red): Tumor cells; GFP (green): Stromal fibroblasts; DAPI (blue): Nuclei; EdU (pink): Proliferating nuclei.

For the analysis, alginate-BR samples have non-spherical shapes and the automated segmentation step based on ellipsoid approximation is not valid. Therefore it is replaced by a manual delineation of the spheroid contours in 2D (taking the 3D images as a base for examination of the spheroids) and subsequent detection of the spheroid geometric center in 3D. Afterwards, EdU positive cells are detected using the 3D spot detection algorithm from^10^, illustrated in Fig. 3a,b. The cells below the center of the spheroid are ignored and upper and lower parts of the spheroids are considered equivalent.

**Step 1:** Collect alginate capsules from the BR and place them in an 8-well plate (μ-Slide 8 well, ibiTreat). Cover alginate microcapsules with low melting agarose (Sigma) for live image acquisition, or with ProLong Anti-fade for fixed samples, in order to immobilize the capsules.

**Step 2:** Acquire 3D image stacks

*Microscope settings see Table 5.

*Images of at least 10 spheroids for the 3 biological replicates should be acquired. Fields with less than 3 spheroids or fields too close to the border were excluded.

*Image acquisition for size and morphology analysis: Using a lower magnification objective (10x) allows for the acquisition of a representative number of spheroids (3 or more) per field. The limited imaging depth of the objective, however, restricts image acquisition to approximately half of the spheroid. Image acquisition was always initiated from the bottom of the spheroid.

*Image acquisition for proliferation analysis: The 20x magnification objective allowed a visualization of EdU positive cells in the inner parts of the spheroids while still covering whole spheroids in the xy-plane. The z-step is set to 1 μm, to resolve the EdU positive cells. To allow correlation-studies between size, shape and proliferation, all spheroids of the field acquired from the lower magnification (10x) were also imaged with the 20x as sub-images.

**Step 3**: Analysis: Size and 2D shape analysis may be analyzed by the automated analysis algorithm and parameters provided on (Data Citation 1) and entitled 'MATLAB script for the image analysis of 3D image stacks of 3D multi-cellular spheroid cultures'. For the analysis of EdU positive cells, the algorithm input allows manually outlined spheroids and z-location parameters as ImageJ ROI’s.

*Spheroid size determination: Due to the above-described restrictions of imaging depth, spheroid areas rather than volumes should be quantified. It should be noted, however, that area or volume do not necessarily reflect cell growth or death, since cell density may vary considerably between spheroids. Also the addition of stromal cells may affect spheroid compactness e.g., via stiffness and various deposits. EdU or some cell death markers may be required if cell proliferation or death is an important readout.

#### 3D matrix-embedded cell cultures

These cultures contain many, relatively small homogenously distributed spheroids with area sizes of up to 0.005 mm^2^. When imaged at the stationary phase, as in our setting, the number of EdU-positive cells is relatively low. The shape of the spheroids depends on the matrix: Matrigel results in nearly spherical spheroids, whereas collagen induces more irregular shapes. The same holds true for combined matrices with different ratios of collagen versus Matrigel (see also ‘Matrix concentration’). Since the spheroids are distributed in three dimensions, the range along the z-direction should be extended as far as possible. All samples should be quality checked for even distribution of spheroids along the z-axis (see validation part), cells growing on the plastic surface, torn/folded ECM because of fibroblast-induced collagen shrinkage or other issues.

3D matrix embedded cell cultures were analyzed using an ellipsoid approximation for the spheroids as described^10^. For samples consisting of very invasive spheroid types (e.g., in collagen), spheroids cannot be approximated by ellipsoids and a less-defined spheroid contour is used instead. Fig. 3c–f illustrates the results for the ellipsoid approximation of a 3D image stack taken from a homogenously distributed assay of prostate cancer cells.

**Step 1:** Place plate under microscope.

**Step 2:** Acquire 3D image stacks:

*For examples of the microscope set-up, see Table 5.

*A compromise needs to be found between resolution, coverage in z-axis, and scan speed/memory usage: We would recommend the use of a 10x magnification objective, which reaches a depth of about 1 mm along the z-axis, with a z-step of 10 μm. This roughly corresponds to the diameter of a nucleus, and thus avoids missing the EdU signal, while optimizing scan-speed. If spheroids contain a large percentage of positive cells, objectives or microscopy set-ups with a smaller axial resolution would have to be used.

*QC checks: Homogeneous distribution, density of the spheroids in the culture (see also technical validation), no cell growth on the bottom. Morphology of the spheroids and consequences for the analysis algorithms (e.g., is the assumption that spheroids are round or oval correct?) Shrinkage or folding/tearing of the matrix.

**Step 3:** Analysis: Spheroids may be analyzed using the automated analysis algorithm and parameters provided on (Data Citation 1) and entitled 'MATLAB script for the image analysis of 3D image stacks of 3D multi-cellular spheroid cultures'.

*Input parameters for the automated spheroid segmentation have to be adjusted according to the expected spheroid shape—spherical to invasive morphology (see also the images on (Data Citation 1) where example images for each of this types are provided).

*Spheroid homogeneity and density may be assessed post-analysis.

#### Formalin-fixed paraffin-embedded (FFPE) 3D, tissue slice cultures

**Figure 4:**
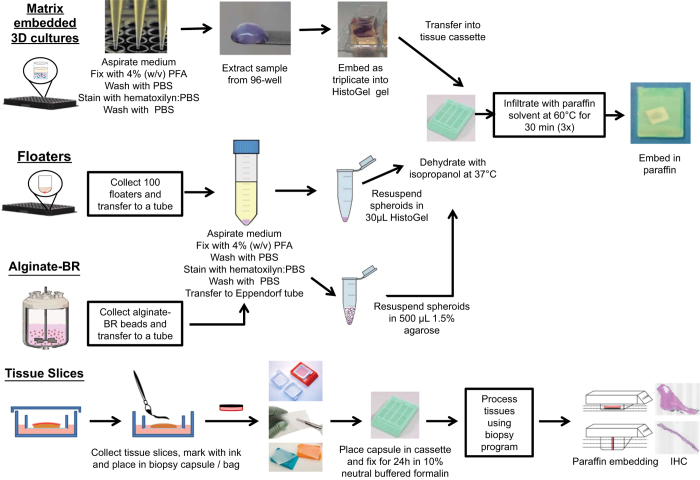
Flow diagram illustrating the steps of processing 3D culture models. Collagen or collagen/Matrigel embedded 3D cultures, floaters, alginate-BR, and tissue slices.

Being able to generate paraffin embedded blocks of 3D cultures and tissue slices is essential for the analysis of the structural and functional complexity of a given model, particularly its heterogeneity. The spatial configuration of cells, as well as their interactions with neighboring cells and the extracellular matrix are preserved. Moreover, paraffin blocks may be stored for long periods of time, remaining accessible and allowing multiple researchers to address their model-specific questions. Within the PREDECT project, protocols to process complex 3D cultures and tissue slices for paraffin embedding were established, allowing for a cross-comparison of the models using TMAs (tissue microarrays). These methods were briefly described in the original manuscripts^5,9^; the individual steps are described and illustrated in more detail below and in Fig. 4.

#### 3D floater cultures

The protocol starts from floaters cultivated in U-bottom 96-well (Corning #4520) or 384-well plates (Corning, # 3830).

**Step 1**: Collect ca. 100 spheroids into a Falcon tube.

**Step 2**: When all spheroids have settled to the bottom of the tube, aspirate medium and wash the spheroids in PBS twice, taking great care not to lose the spheroids, before fixing for 20 min in 5 ml of 4% paraformaldehyde. Wash again twice in PBS, stain for 10 s in a 1:1 solution of Mayer’s Hematoxilyn:PBS and wash another three times in PBS.

**Step 3:** Heat a tube of solid HistoGel (ThermoScientific, HG-4000-012) in a dry block heater (ThermoScientific, 2050Q) to 60 °C. Resuspend the spheroids in 1 ml PBS and transfer them to a 1.5 ml tube. Remove the supernatant carefully, then add 30 μl of HistoGel to the spheroids, and mix the solution in order to homogenously distribute the spheroids within the HistoGel. Let the HistoGel set by cooling the tube to 4 °C for 15 min.

**Step 4–6:** Remove the slide of the 8-chamber, wrap the now solid plug into histo-wrap paper and place it into a tissue cassette for automatic processing.

*If shrinkage of the sample during dehydration is an issue, the samples may be processed manually, following the same protocol as for the automatic processor, i.e., 20 min in 50% isopropanol, twice 20 min in 70% isopropanol, twice 30 min in 95% isopropanol and three times 30 min in 100% isopropanol at 37 °C. Also for the manual protocol, washing with paraffin solvent followed in the tissue processor at 60 °C, three times 30 min, with vacuum and pressure off.

Finally, embed the samples in paraffin wax.

#### 3D alginate-BR cultures

The protocol starts with a stirred tank culture of alginate microencapsulated spheroids, with and without fibroblasts.

**Step 1**: Collect the microencapsulated spheroids into a Falcon tube.

**Step 2**: Let the microcapsules settle to the bottom of the tube, then aspirate the medium and wash the spheroids carefully with PBS twice, taking great care not to lose the spheroids. Fix them for 20 min in 5 ml of 4% paraformaldehyde/4% sucrose solution. Wash again twice in PBS, stain for 10 s in a 1:1 solution of Mayer’s Hematoxilyn:PBS and wash another three times in PBS.

**Step 3:** Boil a 1% (w/v) agarose (Lonza) solution to dissolve the agarose, then let the agarose temperature cool down to 50 °C (in a water bath or a thermomixer). Resuspend the microcapsules in 1 ml PBS then transfer them to a 1.5 ml tube. Remove the supernatant carefully, resuspend the microcapsules with 50 μl of the agarose solution, and mix well in order to homogenously distribute the spheroids within the gel. Let the agarose cool down on ice for 5 min.

**Step 4–6**: As described above.

#### 3D matrix-embedded cultures

This procedure does not work with pure Matrigel since the fixation step dissolves the gel.

Multiple samples may be processed at the same time.

The protocol starts with a 96-well plate of matrix-embedded cultures.

**Step 1**: Carefully remove the medium supernatant, and fix the samples by filling the wells with 4% paraformaldehyde. After 20 min at RT, wash the wells carefully with PBS, and stain the samples with a 1:1 solution of Mayer’s Hematoxilyn:PBS for 10 s and wash again with PBS.

*The latter step enables an easy localization of the spheroids within the paraffin block later.

**Step 2**: Extract the samples from the well using a lab spatula.

**Step 3**: In order to maintain the integrity of the fragile 3D cultures throughout the remaining embedding protocol, the samples should be pre-embedded in HistoGel (ThermoScientific, HG-4000-012).

*Optimal results were obtained when three 3D spheroid-matrix plugs (a triplicate) were combined.

Heat a tube of solid HistoGel in a dry block heater (ThermoScientific, 2050Q) to 60 °C, and pre-coat a chamber of an 8-chamber slide (Labtek #155411) with 100 μl of the now liquid HistoGel. Place the triplicate spheroid-matrix plug on top of the pre-coating and overlaid it another 300 μl of HistoGel. Then transfer the 8-chamber slide to 4 °C for 15 min for the Histogel to set.

**Step 4–6**: As described above.

#### Tissue slice cultures

**Step 1:**Remove tissue slice carefully from the organotypic support using a spatula and probe. Millipore supports are non-adherent, allowing slices to be removed easily.

*Not all filter supports are non-adherent—if FFPE processing is planned make sure to choose a non-adherent support or an adherent support that can be processed with the tissue.

**Step 2:** To ensure correct orientation of the slice when analysing samples via IHC, paint the air-side of the slice with marking ink for pathology.

**Step 3:** Place slice into a biopsy capsule (e.g., CellPath, EBE-0201-02A), a biopsy bag, or two layer of filter papers (e.g., R. Langenbrinck,Labor- und Medizintechnik) and place in normal histology cassette to ensure the slice remains flat during processing.

**Step 4:** Fix the slices in formalin for a minimum of 24 h prior to paraffin embedding,

*Normal processing programs can be used but it is also possible to process slices on a rapid 2.5 h program used for biopsies.

**Step 5:** Slices can be embedded horizontally or vertically to capture any changes in biomarker expression

*See validation section for a discussion about the impact of the slice orientation on the experimental readout.

#### Tissue microarray construction

**Figure 5:**
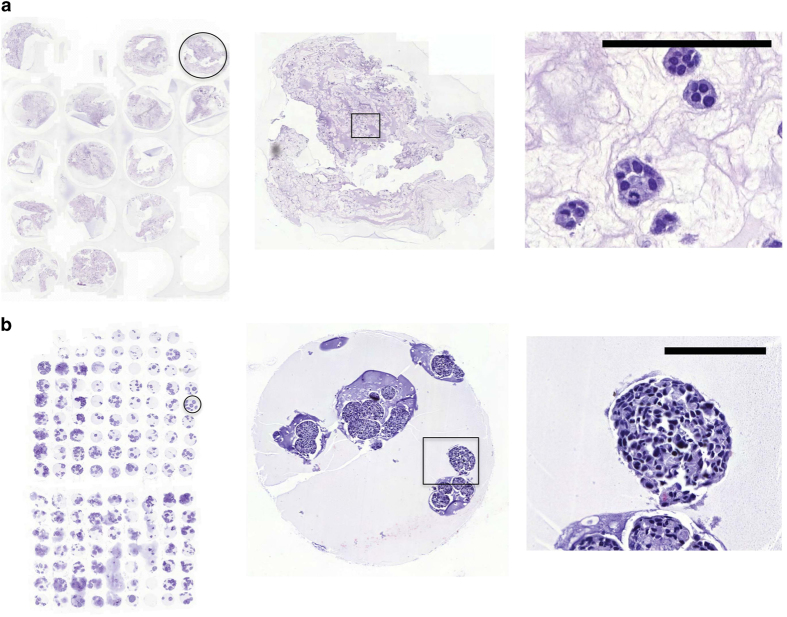
Tissue microarrays and examples of single cores. 5 mm core size for MCF7 as embedded 3D culture (**a**) and 1 mm core size for H1437 cell line cultured in alginate-BR (**b**). Scale bar 100 μm.

All cell culture FFPE blocks were collected and archived centrally at the Institute for Molecular Medicine Finland (FIMM) as tissue microarrays (TMA)^11^. Examples for TMAs and cores are shown in Fig. 5.

**Step 1:** Punch a single core from the FFPE blocks using a semi-automatic punching device (MiniCore, Mitogen, UK).

**Figure 6:**
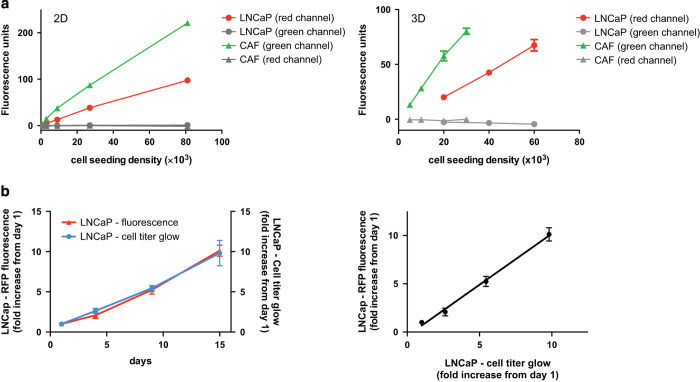
RFP and GFP signals may be used as a proxy to monitor cell growth in 2D and 3D. (**a**) tRFP and eGFP signals correlated with the cell numbers of LNCaP and CAF cells, respectively, when grown in 2D (left panel) or 3D cultures (right panel). LNCaP and CAF cells were seeded at different cell densities (*N*=3–6). One day after seeding the cells, the fluorescent signal was measured at the bottom of the well by spectrophotometer. No bleed-through was observed when tRFP-labeled LNCaP were measured in the green channel (grey line with circles), or eGFP-labeled CAF cells in the red channel (grey line with triangles). Error bars are standard deviation from the mean (*N*=3–6). (**b**) Comparison of fluorescent protein- and Cell titer Glow assay-based measurement of LNCaP 3D spheroid growth. 3D models were set up and growth of LNCaP tumor cells was monitored via RFP fluorescence intensity (left panel/left y-axis) or the cell titer glow assay (left panel/right y-axis), which measures intracellular ATP content, and hence requires cell lysis. Fold increase of fluorescence units (left panel/left y-axis) and luminescence units (left panel/right y-axis) relative to day 1 are shown over the course of 15 days. Error bars represent the standard deviation from mean values (*N*=3). The right panel shows the excellent correlation between the two measurements (r^2^=0.9566; linear regression), suggesting that fluorescent protein-based measurement, which does not require cell lysis, is a reliable method for monitoring 3D culture growth.

*A 1 mm core size was used for cell culture samples with high cell density (2D, floaters and bioreactor cultures). For matrix-embedded 3D cell cultures with low cell density, a core size of 5 mm was selected (Fig. 6).

*If samples are very heterogeneous, and the blocks allow for it, more than 1 punch per sample should be included in the TMA.

*Use an asymmetric design for the array in order to be able to track sample locations

**Step 2:** Cut 3.5 μm sections on Superfrost objective glasses (Kindler O Gmbh, Germany) using, e.g., a Microm 355S microtome (Thermo Scientific, Waltham, MA). Dry slides on heating block or over-night at 37 °C. Store slides at −20 °C for long-term.

#### Immunohistochemistry

**Step 1:** If multiple IHC markers are to be stained, it is advisable to cut all TMA sections in one cutting session to obtain only consecutive sections. Adhere paraffin sections to glass slides overnight at 37 °C.

*Slides may be stored at −20 °C for at least 6 months.

**Step 2:** Deparaffinize sections:

Three times 5 min in Xylene (Fluka/FFChemicals)

Three times 1 min in 99.8% EtOH (Altia)

Twice 1 min in 96% EtOH

Once 1 min in 70% EtOH

Once 1 min in water

**Step 3**^**#**^: Induce antigen retrieval at 99 °C, for 20 min in 10 mM Tris-HCl (Sigma)+1 mM EDTA (Sigma)

*Buffer stable for two weeks or 40 slides

**Step 4**^**#**^: Wash for 3 min in 1X PBS (Biotop)+0.05% Tween20 (Fisher Scientific)

Step 5*: Incubate for 15 min in 0.9% H_2_O_2_ solution (Fluka) to block peroxidase activity

Step 6*: Wash in 1X PBS+0.05% Tween20.

Step 7*: Incubate for 15 min in 1X PBS+10% GNS (Millipore) to block non-specific binding

*Air blow the blocking solution off the slide before primary antibody (do not wash!). If manual protocol is used, pour/tap the blocking solution off the slide.

Step 8*: Dilute primary antibody in 1X PBS+10% normal goat serum (NGS; Millipore).

Incubate for 90 min (or as applicable) at RT

Step 9*: Wash twice in 1X PBS+0.05% Tween20

Step 10*: Dilute secondary antibody (Immunologic) in 1X PBS+10% GNS

Incubate for 30 min at RT

Step 11*: Wash twice in 1X PBS+0.05% Tween20

Step12*: Stain with DAB (3,3′-Diaminobenzidine; Immunologic) according to manufacturer’s instructions

Step 13*: Rinse with water to stop DAB stain reaction

**Step 14:** Counterstain 1 min in 25% Mayers Hematoxylin (DAKO) in H_2_O

**Step 15:** Rinse for 5 min in running cold tap water

*Avoid direct exposure of the slides to the running water

**Step 16:** Dehydrate

Once 1 min in 70% EtOH

Twice 1 min in 96% EtOH

Three times 1 min in 99.8% EtOH

Three times 1 min in Xylene

**Step 17:** Mount using Pertex-mountant (HistoLab), leave to air dry

^#^in a PT Module (Thermo Scientific): Steps 3–4

*in a Lab Vision Autostainer 480S (Thermo Scientific): Steps 5–13

#### Hematoxylin and Eosin staining (H&E)

**Step 1:** Deparaffinize sections:

Three times 5 min in Xylene (Fluka/FFChemicals)

Three times 1 min in 99.8% EtOH (Altia)

Twice 1 min in 96% EtOH

Once 1 min in 70% EtOH

Once 1 min in water

**Step 2:** Incubate 10 min in Mayer´s Hematoxylin (undiluted)

**Step 3:** Flush for 5 min in running cold tap water water (avoid direct exposure of the slides to the running water)

**Step 4:** Differentiate by dipping the slides twice in 70% EtOH+1% HCl

**Step 5:** Flush in running cold tap water, 5 min

**Step 6:** Incubate in 0.5% Eosin, 30 s

**Step 7:** Dehydrate

Twice 1 min in 96% EtOH

Three times 1 min in 99.8% EtOH

Three times 1 min in Xylene

**Step 8:** Mount using Pertex-mountant, leave to air dry

## Data Records

### Generation of growth curves for 2D/3D cultures

Examples for growth curves of 2D (LNCaP (RFP)/WPMY1 (GFP) OR CAF (GFP): RawData_2D_Prostate_SD.xls), 3D floaters (LNCaP (RFP)/WPMY1 (GFP) OR CAF (GFP): RawData_Floaters_Prostate_SD.xls), 3D BR (H1437 (RFP)/NF (GFP) OR CAF (GFP) AND MCF7 (GFP)/NDF (RFP): RawData_BR Lung_Breast_SD.xls), and 3D matrix-embedded cultures (MCF7 (GFP)/NDF (RFP): RawData_3Dmatrix_Breast_SD.xls; LNCaP (RFP)/WPMY1 (GFP) OR CAF (GFP): RawData_3Dmatrix_Prostate_SD.xls; H1437 (RFP)/NF (GFP) OR CAF (GFP): RawData_3Dmatrix_Lung_SD.xls) have been uploaded to (Data Citation 1). The examples are as described in Ref. 5.

### Image analysis of fluorescence-labeled 3D cultures

The following section describes the 3D analysis procedures used for the 3D analysis of the 3D fluorescent spheroid cultures used in Ref. 5. The main tools used for the analysis are:

- FIJI (ImageJ): Used to outline manual ROI's of spheroids (using the RoiManager),
- MATLAB: 3D spheroid segmentation, shape analysis and EdU positive cell detection,
- CellProfiler: 2D spheroid detection and 2D shape analysis,
- R & RStudio: Data analysis of the output of the above two.

We also used ZEN 2.0 LIGHT for the conversion of czi formatted raw image data to tif-stacks.

Below we try to provide a workflow for each of the different fluorescent 3D cultures. The raw image(s) have been uploaded to (Data Citation 1), and the metadata corresponding to the file names of the images is documented in the samples overview table ‘sample_overview_table.xlsx’ which we will refer further to as the ‘samples overview table’. The raw images are either 3D multi-channel stacks taken with a confocal microscope, or 2D (multi-)channel widefield images. The image analysis of the 3D confocal stacks is mostly done using the algorithm provided at https://github.com/mbarbie1/ellipsoids-analysis-paper, while 2D widefield images are analyzed using CellProfiler pipelines. Both are uploaded to (Data Citation 1) as ‘ellipsoids-analysis-paper-master.zip’ (entitled: ‘MATLAB script for the image analysis of 3D image stacks of 3D multi-cellular spheroid cultures’) and ‘Widefield_bioreactor_breast(lung, prostate)_gfp.cpproj’ (entitled: 'Example CellProfiler pipeline for the image analysis of the prostate cancer cell floater cultures').

The data output from the MATLAB script ‘ellipsoids-analysis-paper-master.zip’ contains 3 tables: msrSpheroids.csv, msrSpots.csv and summary.csv, and is described in the file ‘output_data_ellipsoid_analysis_description.xlsx’ which is also uploaded to (Data Citation 1) and entitled: ‘MATLAB script: overview of the output’. To use the CellProfiler pipelines, the location of the image files inside the CellProfiler pipeline should be adapted to one's own needs as well as the pattern to extract the image metadata from the files (this can be done in the input modules ‘Images’ and ‘Metadata’). The default output folder in CellProfiler is used to save the output, but this can also be adjusted in the SaveImages and ExportToSpreadSheet modules if desirable. The input images are assumed to be RGB images, from which then the different channels are extracted, if the separate channels are available, it is probably better to use those and to adapt the pipeline accordingly.

#### Bioreactor samples

Samples originating from the bioreactor, referred to as BR-alginate in the samples overview table, were imaged both with widefield and confocal microscopy. The workflow for both is described below.

#### Widefield images

The widefield images are analyzed using the CellProfiler pipeline in ‘Widefield_bioreactor_lung_rfp.cpproj’ and ‘Widefield_bioreactor_breast_gfp.cpproj’, which are entitled: ‘Example CellProfiler pipeline for the image analysis of the breast(Iung) cancer cell bioreactor cultures’. As explained in the introduction the CellProfiler pipelines should be adapted to one's own needs.

#### Confocal stack images

For the confocal stack images first the 3D spheroids in the images can be manually outlined using the RoiManager of FIJI. These ROI’s can then be provided to the analysis script in the MATLAB procedure at https://github.com/mbarbie1/ellipsoids-analysis-paper. ROI’s must only be provided when the automated spheroid detection cannot discern different spheroids, which can happen when the imaging depth is less than the spheroid size.

#### Floater samples

The floaters are imaged using widefield microscopy, and can be analyzed by simple CellProfiler pipelines provided in ‘Widefield_floater_breast_gfp.cpproj’, ‘Widefield_floater_prostate_rfp.cpproj’, and ‘Widefield_floater_lung_rfp.cpproj’, which are entitled: ‘Example CellProfiler pipeline for the image analysis of the breast(Iung, prostate) cancer cell floater cultures’. As explained in the introduction the CellProfiler pipelines should be adapted to one's own needs.

Treated floater samples tended to be extremely fragile and could not be processed for imaging.

#### Matrix-embedded samples

For the confocal stack images of matrix embedded cultures the analysis script in the MATLAB procedure in ‘ellipsoids-analysis-paper-master.zip’ can be used without manually outlining the spheroids.

The examples uploaded to (Data Citation 1) are as described in Ref. 5. Due to their size, only a selection of the embedded prostate imaging stacks has been uploaded.

## Technical Validation

### Tracking cell growth via RFP/GFP fluorescence

Tumor and stromal cell lines were transduced with lentiviral constructs, encoding either red fluorescent protein (RFP) or green fluorescent protein (GFP). This reporter system, if truly reflecting the number and viability of cells in a culture, enables live-cell monitoring of tumor and stromal cell growth in parallel in the same culture. However, as described below, the correlation with cell growth should be established for every system using (an) independent modalit(y/ies).

#### GFP and RFP signals do not overlap

In order to make sure the fluorescence proteins were specific for tumor and stromal cells, respectively, increasing numbers of cells were plated in 2D, and the fluorescence signal measured using filter sets for either fluorescent protein.

No bleed-through was observed from one fluorescence channel into the other, and a positive correlation between RFP/GFP signals with cell numbers could be demonstrated (Fig. 6a). Similar correlations were observed when increasing number of cells were embedded into 3D matrix and fluorescence was measured the next day (data not shown).

#### Fluorescent- and metabolism-based growth assays correlate

In order to ensure that the fluorescence signal detected from GFP or RFP reflected actual cell growth, Cell Titer Glo (Promega), which is based on a measurement of the cells’ ATP content, was chosen as an alternative means to quantify the increase of cell mass in 3D cultures. LNCaP cells were cultivated as floaters, and growth of the cultures was measured in parallel at the same time points.

Growth could readily be measured with either assay. Both measurements showed similar growth characteristics, confirming that the fluorescent protein-based measurement was a reliable method for live-cell monitoring of the growth of 3D cultures (Fig. 6b).

As a second standard cell viability assay, Alamar blue, which is converted to a red-fluorescent dye, resorufin, in cells with intact mitochondria, was compared to RFP fluorescence for its potential to detect Paclitaxel-induced cell killing. LNCaP cells were embedded into matrix and treated with increasing doses of the cytotoxic agent Paclitaxel. After 24 h, the RFP fluorescence signal was measured, before Alamar blue was added, and, 3 h later, the fluorescence of the generated resorufin was detected.

The dose response curves of both measurements showed similar trends, indicating that the fluorescent reporter signal-based measurement of cell viability was comparable to a standard cell viability assay (Supplementary Fig. S3). Thus, the reporter signal-based measurement should be a reliable method to establish relative cytotoxicity of agents in drug treatment studies.

As a third alternative assay, Picogreen (Quant-iT PicoGreen dsDNA Assay; Kit P7589; Thermo Fisher Scientific) was tested in the Alginate-BR system. Picogreen is based on the fluorescent quantification of a probe that intercalates into dsDNA. This assay was chosen as a validation method since the DNA concentration is independent of the differentiation state and identity of a cell, contrary to total protein concentration.

1 ml of culture suspension was collected every 3–4 days, in duplicates, and the DNA was quantified using the Picogreen protocol, according to the manufacturer’s instructions. Fluorescence measurements were performed in the fluorescence reader (Table 5). Results are presented as a fold increase compared to day 1 (Supplementary Fig. S4).

The fluorescent protein-based measurement has several advantages over the classical metabolism based assays, since it allows, (1), for real-time growth monitoring of the spheroids and, (2), in co-cultures, for parallel measurements of tumor and, e.g., stromal cells. The only requirement is that the cells’ fluorescence be bright enough for detection by the spectro-fluorometer.

### 3D matrix embedded culture set-up

Conditions for 3D embedding have to be optimized for any mono- or co-culture experiment. This section describes the optimization process for the PREDECT 3D embedded cultures. Several parameters, such as cell numbers, ratios, and matrix density, were tested.

#### Cell density

**Figure 7:**
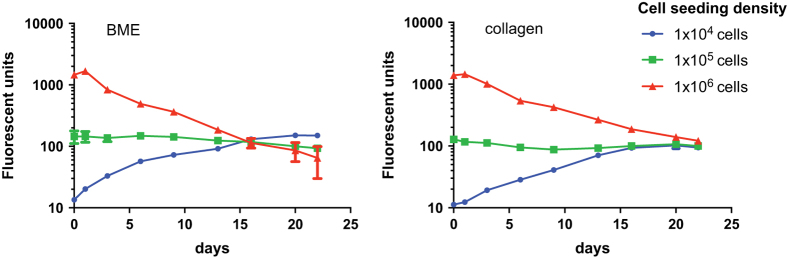
Cell density titration in BME and collagen. LNCaP tumor cells were seeded as triplicates in Matrigel (7 mgml^−1^ (BME/Trevigen); left panel) or collagen (right panel) at densities 1×10^4^, 1×10^5^, and 1×10^6^ cells per well. Growth of LNCaP tumor cells was monitored via RFP fluorescence, shown as mean absolute fluorescent units with error bars marking the standard deviation (*N*=3). Seeding 1×10^4^ cells resulted in exponential growth in both matrices (blue), 1×10^5^ cells remained in equilibrium (green) while 1×10^6^ cells appeared too dense for growth or cell survival to be supported (red).

The first step was to optimize the number of tumor cells to be seeded in 90 μl matrix per 96 well. As shown in Fig. 7, seeding 10,000 tumor cells per well resulted in an exponential growth in both basement membrane extract (BME, Trevigen) and collagen I (BD Bioscience), reaching a plateau after approximately 14 days. When increasing the number to 100,000 tumor cells, the cell density remained constant over the course of the experiment. When increased to 1,000,000 cells, the fluorescence signal decreased over time, indicating that this number of cells could not be supported by the matrices. Thus a starting point for our 3D cultures would be 10,000 cells per 90 μl matrix.

**Figure 8:**
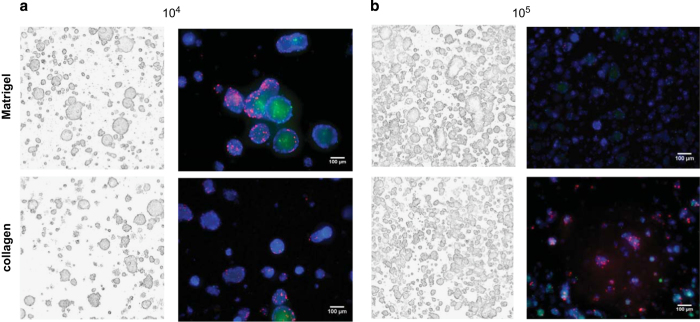
Effect of cell density on spheroid morphology. LNCaP tumor spheroids seeded at 10,000 (**a**) or 100,000 cells (**b**) in Matrigel/BME (top) and collagen (bottom). Maximum projection images on day 14 (left panel) and stained spheroids on day 22 (right panel). EdU (red proliferating cells), NucView (green apoptotic cells), and Hoechst (blue nuclei). Note that larger spheroids have an apoptotic core and more proliferating cells at the outer layers.

The above growth curves suggested that both the 10,000 and 100,000 cell cultures are viable but maximum projection images at day 14 revealed morphological differences. A higher cell density appears to result in smaller spheroids in both BME and collagen (Fig. 8a). Additional proliferation and apoptosis staining on day 22 revealed that both the larger and smaller spheroids have proliferating cells after long term culturing but only the larger ones have an apoptotic core and more proliferating cells on the outer layer of the spheroids (Fig. 8b). Seeding 10,000 cells also resulted in more round shaped spheroids compared to the high-density culture.

#### Matrix concentration.

In order to determine the optimal Matrigel (Corning) concentration to sustain homogenous 3D growth of tumor cells, a Phaedra analysis as described by Cornelissen and colleagues^12^ was performed for a range of Matrigel concentrations from 1–8 mgml^−1^.

**Figure 9:**
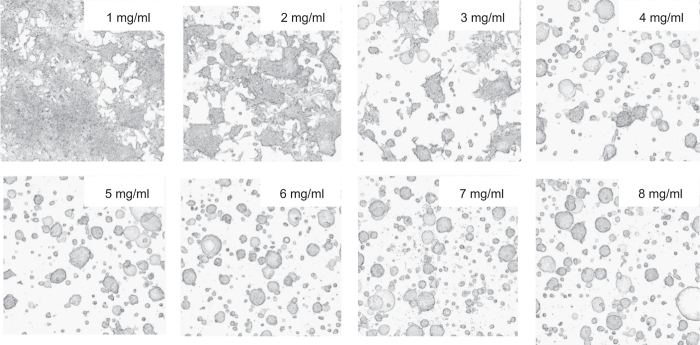
Phaedra analysis of 3D embedded LNCaP tumor cells in Matrigel. LNCaP cells were embedded in 1–8 mgml^−1^ Matrigel. Note that at matrix concentrations of 3 mgml^−1^ or less, cells tend sink to the bottom of the well. Note that at concentrations of 4 mgml^−1^ or higher, an even distribution of the spheroids in the matrix is observed. Also, spheroids in 4 mgml^−1^ Matrigel and higher appear to be more rounded.

The analysis showed that Matrigel concentrations higher than 3 mgml^−1^ resulted in a homogenous distribution of 3D spheroids throughout the gel, whereas lower concentration couldn’t prevent the spheroids from sinking towards bottom of the wells, allowing for 2D growth (Fig. 9). Maximum projection analysis also showed that the 3D spheroids lose their round shape in Matrigel concentrations below 4 mgml^−1^ (Fig. 9). Based on these results and with respect to the cost effective use of Matrigel, the concentration of 4 mgml^−1^ was chosen as appropriate and used in further experiments.

#### Tumor:Stroma cell ratio.

In order to determine the optimal ratio of tumor to stroma cells for 3D embedded cultures, a series of ratios of the two cell types was tested for the growth stimulating effect of the stromal cells on the tumor cells in collagen (here: HDF human dermal fibroblasts and MCF7 breast cancer cells), and the collagen contracting activity of the HDFs. The latter interferes with most downstream analyzes, and should therefore be avoided by limiting the number of stromal cells in a co-culture model.

While a ratio of tumor:stroma cells of 1:1 clearly gave the most pronounced growth stimulatory effect on the MCF7, it also resulted in a strong contraction of the collagen gel (Supplementary Fig. 5). All other ratios gave a similar stimulation of MCF7 growth, except for a ratio of 20:1, and only a 2:1 ratio resulted in a similar contraction of the collagen gel. Thus tumor:stroma cell ratios between 3:1 and 10:1 are functional without affecting the collagen matrix.

### FFPE embedding

Good quality H&E and IHC images from histological sections of a TMA (see ref. 5) are proof of the quality of the FFPE procedure described here (modified from refs 13,14).

#### 3D culture models

We successfully processed tiny, viscous, and fragile 3D cultures, preserving structural complexity and morphological features, except for Matrigel embedded 3D cultures. The structure of 3D complex cultures grown in Matrigel was lost while fixed in 4% paraformaldehyde (not shown). We managed to partly prevent depolymerization of Matrigel by addition of 0.25% glutaraldehyde (Supplementary Fig. S6). The increased aldehyde concentration which appears to provide Matrigel preservation may, however, interfere with antibody binding^15^. Therefore, Matrigel embedded 3D cultures were not included in immunohistochemical analysis.

#### Tissue slice models

Tissue slices represent biologically complex cell-matrix structures of finite depth. Hence, to evaluate the tissue slice composition and biomarker expression across the slice, 10% neutral buffered formalin-fixed slices can either be embedded in paraffin in vertical or horizontal orientation (Supplementary Fig. S7). The choice of orientation depends on the experimental question; the horizontal slice surface analysis allows a larger area of analysis across a single tumor lesion, while the vertical orientation permits analysis of the slice interior, to study potential culture-induced biomarker gradients across the depth of the slice (see next section for further details).

### Validation of tissue slice culture methods

Methods for culturing tumor tissue slices have been described previously, but prior to our publication^9^, these methods had not been compared side-by-side, using the same tumor model and different tumor types. The major advantage of tumor tissue slice culture is the ability to culture primary human tumors from established xenografts or directly from patient material with a minimum of disruption and processing of the tumor. For practical reasons and to robustly validate multiple culture conditions, the PREDECT consortium chose to use largely xenografted tumors from well-characterized human cell lines (CDX), transplantable patient-derived xenografts (PDX), or GEMM-derived tumors, all as surrogates for human primary tumors. By providing an abundance of ‘biologically similar’ tissue, these models allowed for repeat experimentation and tight control of sampling technique important for the optimization and validation process that could not have been easily met if primary human tumors had been used.

Below, some approaches are described that were implemented to monitor the impact of culture methods on tissue viability and integrity, and to analyze the impact of cultivation on tumor cell biology. Tumor slices were FFPE embedded and stained by IHC to assess a variety of biomarkers and the induction of stress markers was assessed by analysis of gene expression.

#### IHC-based qualitative analysis

The impact of two parameters, mechanical support and physiological oxygen levels, were tested in the first instance. For the former, tumor slices were cultured either immersed in media, ‘floating’, or at an air-liquid interface using an organotypic filter support, ‘filter’. For the latter, slices were cultivated either at physiological (3–4%, low, L) or atmospheric (21%, A) oxygen concentrations. A basic pathological examination of H&E stained sections of FFPE-embedded tissue slices, combined with a semi-quantitative assessment of biomarker expression by IHC, was used to evaluate the cultivation parameters.

The use of an organotypic filter support was shown to be superior to the ‘floating’ method, across all tumor types tested. Features of the ‘*in vivo*’ parental tumor were maintained with greater fidelity using the ‘filter’ method (Supplementary Fig. S8).

Whereas the H&E staining clearly showed that cell viabilities in MCF7 CDX and lung GEMM tumors were retained only in atmospheric oxygen conditions, the results were less conclusive for the prostate PC295 PDX, and the lung H1437 CDX, and lung 1,647 PDX models. A quantitative gene expression-based approach (described below) was therefore applied to assess the optimal oxygen levels across the range of models.

#### Gene expression-based quantitative analysis

In order to monitor the viability and functionality of the cultured tissue slices at a molecular level, a set of genes containing key stress and viability biomarkers was assembled (full gene list with annotation is included in the Supplementary Materials). Expression of the selected genes was analyzed from slices cultured at various conditions, and compared to parental tumors, that had been snap-frozen at necropsy, and to a tissue slice frozen immediately after slicing (d0 slice).

Very few gene expression changes were induced by the act of tumor tissue slicing (Supplementary Fig. S9). Amongst the cultured slices, the lowest number of stress genes was induced in tumor tissue slices cultivated in atmospheric oxygen and on a filter support. Thus this condition was not only optimal for tissue viability of MCF7 and the lung GEMM model (see above), but also the prostate PC295 PDX, and lung H1437 CDX and 1,647 PDX tumor models. In line with the histopathological observations, gene changes increased across the models when cultured without a filter support, or with a filter support but at low (physiological) oxygen conditions (Supplementary Fig. S9). Euclidean distance scores, generated by comparing the gene expression values of cultured slices with the gene expression values of the *in vivo* parental tumor, showed that slices cultured in atmospheric oxygen using a filter support were more representative of the *in vivo* situation (Supplementary Fig. S10).

#### Loco-regional biomarker expression across slices

**Figure 10:**
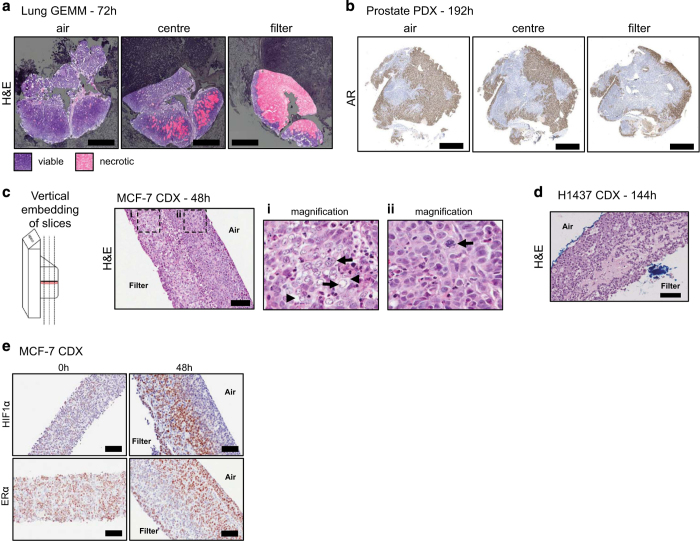
Loco-regional changes in IHC biomarkers in tumor slices cultivated under optimal conditions (filter support, atmospheric oxygen). (**a**) Lung GEMM tumor slices, horizontally embedded, were found to contain large areas of necrosis at the filter interface of the slice. Scale bars represent 1,000 μm. (**b**) AR staining revealed that AR positive cells were in abundance at the air interface in prostate PDX tumor slices and were greatly reduced at the filter interface. Scale bars represent 1,250 μm. (**c**) H&E stained sections of vertically embedded MCF7 CDX tumor tissue slices. Images show differences in tissue morphology dependent on their proximity to the air interface of the slice. (i) Arrows: Areas of necrosis; arrowheads, vacuolated regions (ii) Arrow: Mitotic figure. (**d**) H&E stained section of vertically embedded H1437 CDX tumor slice, showing similar morphology to that observed in MCF7 tumor slices. (**e**) Immunohistochemical staining of HIF1α in MCF7 tumor slices showed an accumulation of nuclear protein at the filter interface of the slice, and immunohistochemical staining of ER, which showed a reduction in staining at the filter interface. Unless specified, all scale bars represent 100 μm. Taken from ref. 9.

In most studies, if histological analysis was performed on tumor tissue slices, the analysis was done on the upward-facing surface of the slice (i.e., the air-interface of the filter supported slices). In order to assess the accuracy of the optimized tissue slice protocol to capture and maintain tumor heterogeneity more broadly, biomarker expression was examined also across the depth of the tissue slice. To this end, tumor slices were embedded horizontally to be sectioned from the air-interface towards the filter side, or vertically and sectioned to incorporate filter- to air-side within one FFPE section (Fig. 10). Horizontal sectioning of lung GEMM tissue slices revealed that viability of the sections decreased with the distance to the air-side (Fig. 10a). Also AR expression in a prostate PDX model decreased in sections closer to the filter support (Fig. 10b). When sectioned vertically, the analysis confirmed a bias in the expression of key biomarkers within each tissue slice with a propensity for viability markers to be found closer to the air-interface, while stress biomarkers (such as HIF1α) were found at the filter-interface (Fig. 10c–e). This led us to conclude and advise that a cross-section through the tissue slice (preferably by vertically embedding) should be examined to allow proper evaluation of tissue slice quality and to assess changes in biomarker expression.

Overall, the findings of these validation approaches suggest that oxygen levels and access to oxygen appear to the main contributory factors to cell viability upon culture of tissue slices. This is likely reflecting the fact that the majority of xenografted or GEMM tumors exhibit a proficient oxygen supply, through extensive and often immature vasculature, which is lost upon excision from the murine host.

## Usage Notes

The use, advantages and disadvantages of the 2D/3D models and tissue slices have been discussed elsewhere^5,9^. Table 6 is, therefore, focused on a comparison of tissue slices and 2D/3D models.

However, all models described in this report are subject to changes and improvements, so our current assessment may change over time.

## Additional information

**How to cite this article:** de Hoogt, R. *et al.* Protocols and characterization data for 2D, 3D, and slice-based tumor models from the PREDECT project. *Sci. Data* 4:170170 doi: 10.1038/sdata.2017.170 (2017).

**Publisher’s note:** Springer Nature remains neutral with regard to jurisdictional claims in published maps and institutional affiliations.

## Supplementary Material



Supplementary Figures

Supplementary Table S1

## Figures and Tables

**Table 1:** Microwell plates used for plate based ‘static’ PREDECT culture models.

**Culture model**	**Plate type**	**Manufacturer**
**2D**	Black 96-well clear flat bottom	*e.g.,* Greiner Bio One #655-088
**3D matrix embedded**	Black 96-well clear flat bottom	*e.g.,* Greiner Bio One #655-088
**3D floaters**	Black 384-well ultra-low attachment clear round bottom	*e.g.,* Corning #3830
Note: for tissue slices, any microwell plates of appropriate volume may be used since slices will not adhere directly to the plate surface.		

**Table 2:** Experimental 2D/3D cell culture conditions used in the PREDECT study.

	**2D**			**3D floater**			**3D embedded**			**3D bioreactor**			
	**TC (/well)**	**TC:SC**	**FBS (%)**	**TC (/well)**	**TC:SC**	**FBS (%)**	**TC (/well)**	**TC:SC**	**FBS (%)**	**TC (/ml)**	**TC:SC**	**FBS (%)**	**Medium**
**Breast**	6,000	03:01	10	1,000	03:01	10	10,000	10:01	2	20,000	01:01	10	DMEM
**Prostate**	10,000	10:01	2	1,000	10:01	2	10,000	10:01	2	N/A	N/A	N/A	RPMI
**Lung**	10,000	10:01	10	400/200*	01:01	10	10,000	10:01	2	20,000	01:01	10	RPMI
TC: tumor cells; SC: stromal cells.													

*mono/co-cultures; N/A: Not applicable (not performed). Taken from ref. 1.

**Table 3:** Set-up of plate readers for real-time growth monitoring.

**Plate Reader**		**Filters**		
**Instrument**	**Label**	**Excitation (nm)**	**Cut-off**	**Emission (nm)**
**BioTek (Synergy HT)**	GFP	485/20	—	528/20
	RFP	530/25	—	590/35
**Tecan (Infinite M200)**	GFP	488	—	509
	GFP	485	—	535
	RFP*	554	—	581
**Molecular Devices (SpectraMax M2e)**	GFP	540	570	587
	RFP	488	515	525
**PE-Wallach (EnVision 2,100)**	RFP*	531	—	590

*dTomato. Taken, with modifications, from ref. 5.

**Table 4:** Media used for tissue slices prepared from xenografts or GEMM tumor models.

**Tumor**	**Media Constituents**
**MCF7**	DMEM (Sigma) supplemented with glutamine (2 mM; Gibco), penicillin (100 Uml^−1^; Gibco), streptomycin (100 μgml^−1^; Gibco) and 10% foetal bovine serum (FBS; Gibco).
**NCI H1437, PDX 1,647**	RPMI −1,640 (Biochrom) supplemented with glutamine (2 mM; Gibco), penicillin (100 Uml^−1^; Gibco), streptomycin (100 μgml^−1^; Gibco) and 10% foetal bovine serum (FBS; Gibco)
**PC295, PC310, PC346C**	DMEM/F12 medium (Lonza, Belgium) supplemented with 2% FBS (Gibco), 1% insulin-transferrin-selenium (Gibco BRL), 0.01% bovine serum albumin (Boehringer Mannheim), 10 ngml^−1^ epidermal growth factor (Sigma-Aldrich), penicillin/streptomycin antibiotics (100 Uml^−1^ penicillin, 100 μgml^−1^ streptomycin; Lonza) plus the following additions: 100 ngml^−1^ fibronectin (Harbor Bio-Products), 20 μgml^−1^ fetuin, 50 ngml^−1^ cholera toxin, 0.1 mM phosphoethanolamine, 0.6 ngml^−1^ triiodothyronine, 500 ngml^−1^ hydrocortisone and 0.1 nM synthetic androgen R1881 (all from Sigma-Aldrich)
**Kras**^**G12D**^**;Lkb1**^**fl/fl**^ **NSCLC GEMM tumor**	F12 (Gibco) supplemented with 100 Uml^−1^ penicillin, 100 μgml^−1^ streptomycin (Gibco), 2 mM glutamax (Gibco), and 22 mM glucose (Sigma-Aldrich) and with 10% fetal bovine serum (Gibco).

**Table 5:** Microscopes, Objectives, and filters for imaging.

**Fluorescence Microscope settings**					
**Platform**	**Instrument**	**Objective (NA)**	**Fluoro-phore**	**Filters**	
				**Excitation [nm]**	**Emission [nm]**
3D floaters	Zeiss (LSM700)	10x EC PLAN NEOFLUAR M27 (0.3)	dTomato	BP 546/12	BP 575–640
			GFP	BP 450–490	BP 515–565
			Hoechst	365	BP 445/50
			EdU	BP 640/30	BP 690/50
	Zeiss (LSM510 META Multiphoton)	10x Plan Apochromat (0.45)	tRFP	561	BP 565–615
			GFP	488	BP 500–530
			Hoechst	405	BP 390–465
			EdU	640	BP 650–710
	ZEISS (Axiovert 200M)	10x A-Plan (0.25)	dTomato	BP 546/12	BP 575–640
			GFP	BP 475/40	BP 530/50
3D BR	Andor (Revolution WD Spinning Disk)	10x Plan Fluor (0.3) 20x Plan Fluor (0.75)	dTomato	561 (DPSS)	BP 595/50
			GFP	488 (OPSL CW)	BP 525/50
			Hoechst	405	BP 460/50
			EdU	640 (DPSS)	BP 685/40
3D matrix	Zeiss (LSM700)	10x EC PLAN NEOFLUAR M27 (0.3)	dTomato	BP 546/12	BP 575–640
			GFP	BP 450–490	BP 515–565
			Hoechst	365	BP 445/50
			EdU	BP 640/30	BP 690/50
	Yokogawa (CV 7,000 Spinning Disk)	10x UPLSAPO (0.4)	dTomato	561	BP 600/37
			GFP	488	BP 522/35
			Hoechst	405	BP 447/45
			EdU	640	BP 676/29
	Zeiss (Axiovert 200M)	10x A-Plan (0.25)	tRFP	BP 546/12	BP 575–640
			GFP	BP 450–490	BP 515–565
			Hoechst	365	BP 445/50
			EdU	BP 640/30	BP 690/50
NA—Numerical aperture; BP—Band pass; DPSS—Diode-pumped solid-state lasers; OPSL CW—Optically pumped semiconductor lasers continuous wave. Taken, with modifications, from ref. 5.					

**Table 6:** Comparison of 2D/3D models with tissue slices.

**· 2D/3D models**	**· Tissue slices**	**Tissue slices**
ease of use		
level	+ to ++++	− to ++
comment	- from very simple (2D mono cultures) to relatively complicated (matrix-embedded co-cultures)	- from technically complicated (tumor models) to difficult to access (patient samples)
	- growth may be followed in real-time	- some parameters can be followed in real-time.
	- most components readily available	- tumor material from patients can be difficult to access and variable
robustness		
level	++ to ++++	− to +++
comment	- more complex models are less robust	- once parameters are established, mouse tumors are robustly sliced
	- important to quality check all components regularly	- difficult to quality control human patient material, or reproduce results
complexity		
level	− to ++	++ to ++++
comment	- maximal complexity tested here included tumor cells, fibroblasts, and ECM components	- tissue slices encompass the full complement of the local tumor-microenvironment
	- can be increased to include immune cell, vascular, or other tissue components	- the function of individual compartments within the slice cultures over time require testing
applications		
examples	- early drug discovery, target validation, resistance markers	- mode-of- action studies of drug candidates, resistance markers
comment	- complexity of models can be stepwise adjusted to fit purpose	- tumor heterogeneity may require a large number of tests
	- conclusions from test results limited to chosen complexity	- slices from patient tumor samples represent closest possible link to clinic

## References

[d1] Figshare Davies E. J. 2017 https://doi.org/10.6084/m9.figshare.c.3727411

