# Supplementary material for: Protocols and characterization data for 2D, 3D, and slice-based tumor models from the PREDECT project
Source: Sci Data. 2017 Nov 21;4:170170. doi: 10.1038/sdata.2017.170 (PMC5697359; doi:10.1038/sdata.2017.170)

## De Hoogt et al: Supplementary figures

### Figure legends

Figure S1. Layout for setting up 3D floater cultures in a 384-well plate.

*In this example 3 different cell conditions are used; tumor cell monoculture and co-cultures of tumor cells with two different fibroblasts. Avoid seeding cells in outer wells as the medium evaporates quicker. Row A and P and column 1, 23 and 24 are filled with PBS and the columns 2, 21 and 22 are used for negative medium only controls.*

Figure S2. Layout for setting up 3D matrix embedded cultures in a 96-well plate.

*In this example 5 different cell conditions are used: two stromal cell monocultures, tumor cell monocultures, and co-cultures of tumor cells with either of the stromal cells. Avoid seeding cells in outer wells as the medium evaporates quicker. Row A and H are filled with PBS, while the outer columns are used for negative matrix only controls.*

**Figure S3.** Comparison of fluorescent protein-based and Alamar blue assays to detect Paclitaxel mediated cell killing.

*LNCaP cell viability was measured in the same culture comparing a metabolic assay, Alamar blue (left y-axis) with RFP fluorescence intensity (right y-axis). Alamar blue measures mitochondrial activity in live cells, but cannot distinguish between tumor and stromal cells.*

*LNCaP cells were embedded in BME and treated with Paclitaxel for 8 days. Mean fluorescence units of both assays are shown in relation to the paclitaxel concentration*. *Error bars represent the standard deviation of the mean (N=3). Both assays showed a similar dose-dependent decrease of the fluorescence signal upon drug treatment. The IC_50_ values were calculated using a non-linear regression curve fit (Graphpad Prism Software).*

**Figure S4.** Comparison of cell growth monitoring through GFP fluorescence and DNA quantification

*Models were set up in the alginate-BR and growth of MCF7 tumor cells was monitored via GFP fluorescence (left panel), or DNA quantification (Picogreen assay; right panel). Mono and co-cultures were treated with Fulvestrant or Docetaxel. Arrows indicate start of the treatment. Untreated DMSO control curves are indicated with a straight, Fulvestrant with a dotted, and Docetaxel with a broken line. Graphs are shown in green for mono-cultures or in blue for HDF co-cultures. The mean of the fold increase of fluorescence compared to day 1 is shown.* *Error bars represent the standard deviation of the mean (N=4). Both assays showed a similar inhibition of the fluorescence fold increase upon drug treatment. However, only the GFP signal-derived values showed statistically significant decreases on day 20 (unpaired two-tailed students t-test; MCF7 vs MCF7/Fulvestrant: **p=0.0019; MCF7 vs MCF7/Docetaxel: **p=0.0041; MCF7 + HDF vs MCF7 + HDF/Fulvestrant: n.s.; MCF7 + HDF vs MCF7 + HDF/Docetaxel: **p=0.0081).*

**Figure S5.** Ratio of tumor cells:fibroblasts in the MCF7 model in collagen.

*Titration of MCF7 tumor cell: HDF stromal cell ratios in a collagen embedded model. MCF7 and HDF cells were embedded in collagen at ratios of 1:1- 20:1 (tumor:stroma). Growth of the tumor cells was measured via GFP fluorescence intensity. Stromal cell growth could not be detected. Upper panel: Growth curve, showing the mean fluorescence intensity with error bars indicating the standard deviation (N=6). Note the ratio-dependent effect of the stromal cells on the tumor cells. Lower panel: Collagen contraction by HDF stromal cells. At 1:1 and 2:1 tumor:stroma ratios, a marked collagen contraction was detected after 16 days in culture. Taken from Ref 1.*

**Figure S6.** Validation of a fixation method that maintains the integrity of matrix embedded 3D cultures. *Addition of 0.25% glutaraldehyde partially prevented depolymerization of Matrigel observed in 4% paraformaldehyde alone. However, extraction of the gel plug from the well was incomplete. Fixation of cultures in collagen worked with or without (not shown) the addition of 0.25% glutaraldehyde, and extracting the entire collagen plug from the well was not an issue.*

**Figure S7.** Schematic representation of horizontal vs vertical embedding of tumor tissue slices

*(from Ref 2)*

**Figure S8.** Hematoxylin and Eosin (H&E) staining based examination of tissue slice viability.

*Effect of culture conditions (floating vs filter, and atmospheric, A, vs physiological, L) on qualitative tissue morphology features by H&E on the air-interface side of cultured tumor slices from indicated murine sources. Scale bars represent 25µm. Taken from Ref 2*.

**Figure S9.** Induction of stress gene expression dependent on tissue slice cultivation method.

*Total number of changes genes in each culture condition across three models: PC295 PDX, H1437 CDX and 1647 PDX. A – atmospheric; L – low oxygen concentration. Taken from Ref 2.*

**Figure S10**. Euclidean distance scores from stress gene expression analysis.

*Euclidean distance scores determined by hierarchical clustering of the whole gene expression dataset from each model cultured under each condition. Euclidean distances were calculated by comparison to the in vivo parental tumor. Taken from Ref 2.*

### References

1. Stock, K. *et al.* Capturing tumor complexity in vitro: Comparative analysis of 2D and 3D tumor models for drug discovery. *Scientific reports* **6**, 28951, doi:10.1038/srep28951 (2016).
2. Davies, E. J. *et al.* Capturing complex tumour biology in vitro: histological and molecular characterisation of precision cut slices. *Scientific reports* **5**, 17187, doi:10.1038/srep17187 (2015).

### Supplementary Figures

#### Figure S1

#### Figure S2

#### Figure S3

#### Figure S4

#### Figure S5


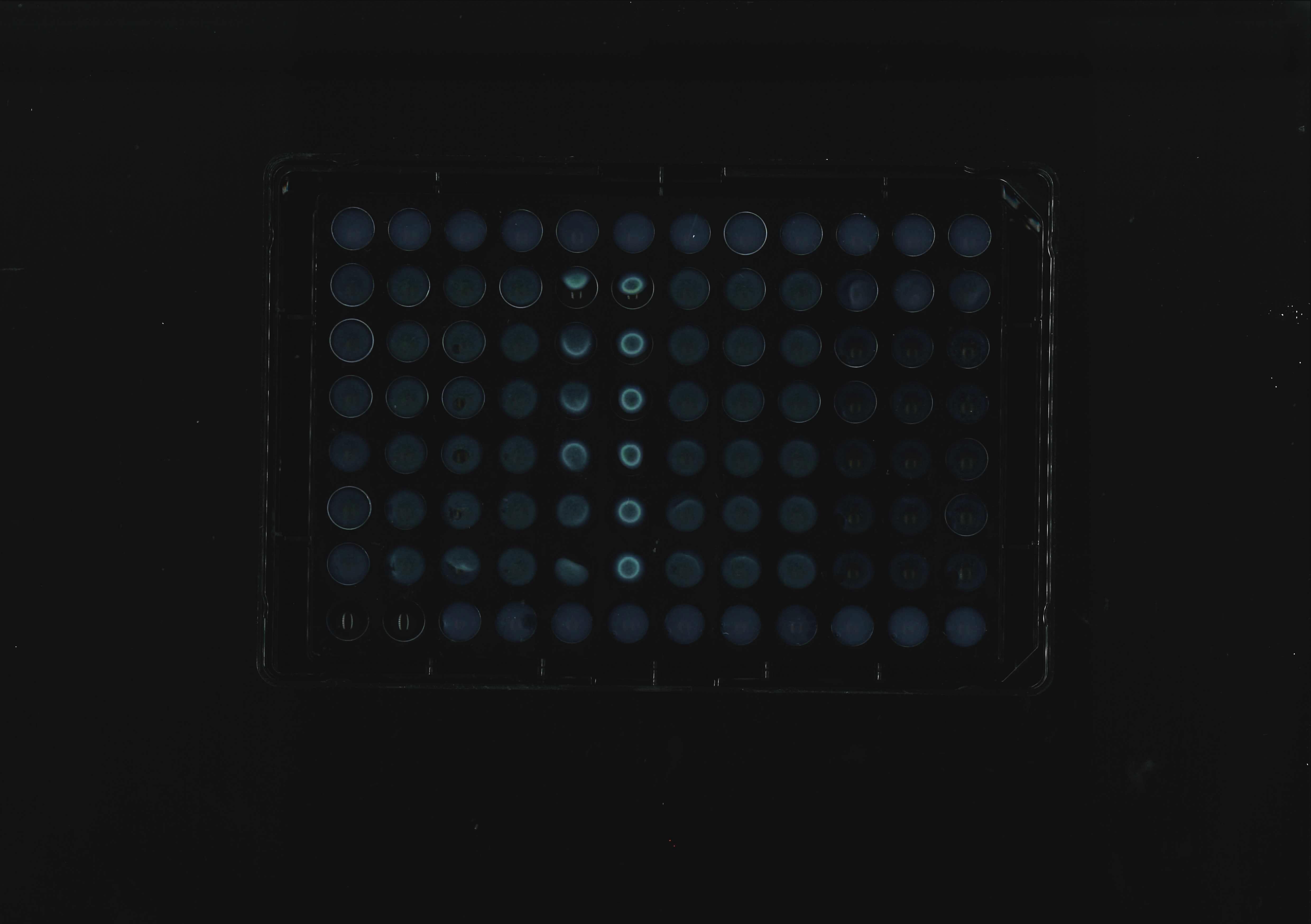

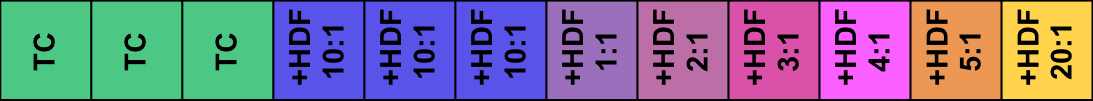


#### Figure S6


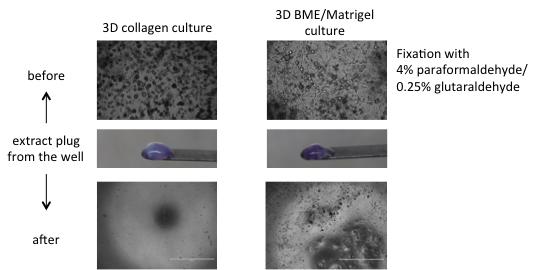


#### Figure S7


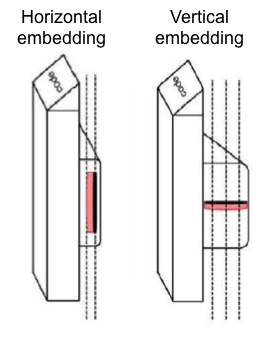


#### Figure S8


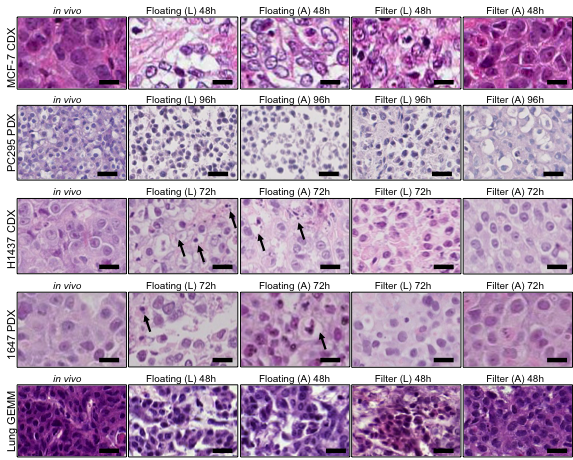


#### Figure S9


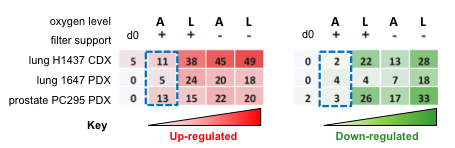


#### Figure S10


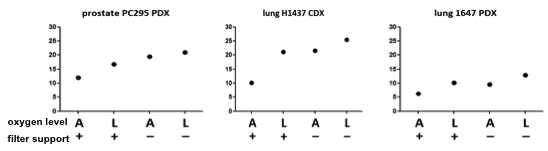

Supplement: Supplementary Figures [file sdata2017170-s2.docx]
