# Supplementary material for: Protocols and characterization data for 2D, 3D, and slice-based tumor models from the PREDECT project
Source: Sci Data. 2017 Nov 21;4:170170. doi: 10.1038/sdata.2017.170 (PMC5697359; doi:10.1038/sdata.2017.170)
Supplement: Supplementary Table S1 [file sdata2017170-s3.docx]

| **Gene names** | **Assay (Applied)** | **Functional Gene Grouping** | **Upstream pathway/transcrition factor** | **Refs #** |
| --- | --- | --- | --- | --- |
| **ABCB1** | **Hs00184500_m1** | **Drug transporter** | p53/NFBkB | ^1^ |
| **ACTB** | **Hs03023943_g1** | **housekeeping gene** |  |  |
| **AIFM2** | **Hs01097299_m1** | **apoptosis** | p53 | ^2^ |
| **ALOX12** | **Hs00911144_m1** | **apoptosis** | NFkB | ^3^ |
| **APAF1** | **Hs00559440_m1** | **apoptosis** | p53 | ^4^ |
| **ATF2** | **Hs01099617_m1** | **UPR/ER stress** | target of stress regulated miR-451 | ^5^ |
| **ATF3** | **Hs00231069_m1** | **UPR/ER stress** | p53/p38 | ^6^ |
| **ATF4** | **Hs00909569_g1** | **UPR/ER Stress** | UPR target gene | ^7^ |
| **ATF6** | **Hs00232586_m1** | **UPR/ER Stress** | UPR transcrition factor | ^8^ |
| **ATG12** | **Hs01047860_g1** | **necrosis/autophagy** | taget of ATF4 | ^9^ |
| **ATG5** | **Hs01555465_m1** | **necrosis/autophagy** | induced by DNA damage | ^10^ |
| **ATG7** | **Hs00197348_m1** | **necrosis/autophagy** | target of miR17 | ^11^ |
| **ATM** | **Hs00175892_m1** | **DNA damage and repair** | BRCA1/E2F1/CtIP | ^12^ |
| **ATR** | **Hs00992132_m1** | **DNA damage and repair** | target of stress regulated miR-185 | ^13^ |
| **BAK1** | **Hs00832876_g1** | **apoptosis** | p53 | ^14^ |
| **BCL2** | **Hs01048932_g1** | **apoptosis** | NFkB/p53 | ^15^, ^16^ |
| **BCL2A1** | **Hs03405589_m1** | **apoptosis** | p53/NFkB | ^17^,^18^ |
| **BCL2L1** | **Hs01067345_g1** | **apoptosis** | NFkB | ^19^ |
| **BCL2L11** | **Hs01083836_m1** | **apoptosis** | NFkB | ^20^ |
| **BCL2L13** | **Hs00209787_m1** | **apoptosis** | prosurvival signals and oncogenes |  |
| **BCL2L14** | **Hs00373302_m1** | **apoptosis** | p53 | ^21^ |
| **BECN1** | **Hs01011594_g1** | **necrosis/autophagy** | NFkB | ^22^ |
| **BID** | **Hs00609632_m1** | **apoptosis** | p53 | ^23^ |
| **BIK** | **Hs00154189_m1** | **apoptosis** | DNA damage/cytokines | ^24^ |
| **BIRC2** | **Hs01112284_m1** | **apoptosis** | NFkB | ^25^ |
| **BLM** | **Hs01119886_g1** | **DNA damage and repair** | prosurvival signals and oncogenes | ^26^ |
| **BNIP3** | **Hs00969289_m1** | **apoptosis** | NFkB | ^27^ |
| **BOK** | **Hs00261296_m1** | **apoptosis** | p53 | ^28^ |
| **BRCA1** | **Hs01556185_m1** | **DNA damage and repair** | regulated by specific factors binding single-stranded DNA | ^29^ |
| **BTG2** | **Hs00198887_m1** | **proliferation/cell cycle regulation** | p53 | ^17^ |
| **CCNA2** | **Hs00153138_m1** | **proliferation/cell cycle regulation** | p53 | ^30^ |
| **CCNB1** | **Hs01030097_m1** | **proliferation/cell cycle regulation** | p53 | ^31^ |
| **CCNG1** | **Hs00171112_m1** | **proliferation/cell cycle regulation** | p53 | ^32^ |
| **CCNK** | **Hs01083210_m1** | **proliferation/cell cycle regulation** | p53 | ^33^ |
| **CDC25C** | **Hs00156411_m1** | **proliferation/cell cycle regulation** | p53 | ^34^ |
| **CDK1** | **Hs00364293_m1** | **proliferation/cell cycle regulation** | p53 | ^35^ |
| **CDKN1A** | **Hs99999142_m1** | **proliferation/cell cycle regulation** | p53/NFkB | ^17^,^36^ |
| **CDKN1B** | **Hs00153277_m1** | **proliferation/cell cycle regulation** | prosurvival signals and oncogenes | ^37^ |
| **CFLAR** | **Hs01116281_m1** | **apoptosis** | NFkB | ^38^ |
| **CHEK1** | **Hs00967510_g1** | **proliferation/cell cycle regulation** | NF-Y-p53 | ^39^ |
| **DDB2** | **Hs03044953_m1** | **DNA damage and repair** | p53/BRCA1/AP1 | ^17^ |
| **DNAJC3** | **Hs00534489_m1** | **UPR/ER Stress** | UPR target gene | ^40^ |
| **EDEM1** | **Hs00976004_m1** | **UPR/ER Stress** | UPR target gene | ^40^ |
| **EDEM2** | **Hs01076556_m1** | **UPR/ER Stress** | UPR target gene | ^41^ |
| **EGFR** | **Hs01076078_m1** | **proliferation/cell cycle regulation** | p53/NFBkB | ^42^,^16^ |
| **EGR1** | **Hs00152928_m1** | **p38/JNK** | NFkB/p38 | ^43^,^44^ |
| **EPHB1** | **Hs01057855_m1** | **UPR/ER Stress** | UPR target gene |  |
| **ERCC1** | **Hs01012156_m1** | **DNA damage and repair** | AP-1/MZF1 | ^45^ |
| **ETS1** | **Hs00901423_m1** | **p38/JNK** | p38 | ^46^ |
| **FANCA** | **Hs01116661_m1** | **DNA damage and repair** | target of miR-503 | ^47^ |
| **FANCD2** | **Hs00395700_m1** | **DNA damage and repair** | transcriptionally regulated under stress (hypoxia) | ^48^ |
| **FANCG** | **Hs00184947_m1** | **DNA damage and repair** | target of stress responsive miR23a | ^49^ |
| **FANCM** | **Hs00913609_m1** | **DNA damage and repair** | HOXC9 | ^13^ |
| **FAS** | **Hs00531110_m1** | **apoptosis** | p53/NFBkB | ^17^,^50^ |
| **FASLG** | **Hs00181226_g1** | **apoptosis** | p53/NFBkB | ^51^ |
| **FEN1** | **Hs01099393_g1** | **DNA damage and repair** | p53/AP1 | ^52^ |
| **FOS** | **Hs01119266_g1** | **p38/JNK** | p53/p38 | ^44^ |
| **FOSL1** | **Hs04187685_m1** | **p38/JNK** | p38 | ^44^ |
| **FTH1** | **Hs01000476_g1** | **ROS** | NFkB | ^53^ |
| **GRB2** | **Hs00257910_s1** | **necrosis/autophagy** |  |  |
| **GSTP1** | **Hs00168310_m1** | **ROS** | NFkB | ^54^ |
| **HERPUD1** | **Hs01124265_m1** | **UPR/ER Stress** | UPR target gene | ^40^ |
| **HGF** | **Hs00900067_m1** | **proliferation/cell cycle regulation** | p53/NFBkB | ^17^,^55^ |
| **HIF1A** | **Hs00936368_m1** | **ROS** | NFkB/transcriptionally regulated under hypoxia | ^56^,^57^ |
| **HMOX1** | **Hs01110250_m1** | **ROS** | NFkB | ^58^ |
| **HRK** | **Hs01388767_g1** | **apoptosis** | prosurvival signals and oncogenes | ^59^ |
| **HSP90AA1** | **Hs00743767_sH** | **UPR/ER Stress** | NFkB | ^60^ |
| **HSPA5** | **Hs00946084_g1** | **UPR/ER Stress** | UPR target gene | ^40^ |
| **IER3** | **Hs04187506_g1** | **apoptosis** | p53/NFBkB | ^16^,^61^ |
| **IGFBP3** | **Hs00426287_m1** | **proliferation/cell cycle regulation** | p53 | ^16^ |
| **JUN** | **Hs00277190_s1** | **p38/JNK** | p38 | ^44^ |
| **JUND** | **Hs02330233_u1** | **p38/JNK** | p38 | ^62^ |
| **KLF6** | **Hs00296661_s1** | **p38/JNK** | p38 | ^44^ |
| **MAFF** | **Hs00202412_m1** | **p38/JNK** | p38 | ^44^ |
| **MAPK8** | **Hs01548508_m1** | **p38/JNK** | KLF4/FOXM1 | ^63^ |
| **MCL1** | **Hs00172036_m1** | **apoptosis** | prosurvival signals and oncogenes | ^64^ |
| **MDM2** | **Hs00234753_m1** | **proliferation/cell cycle regulation** | p53 | ^17^ |
| **MDM4** | **Hs00159092_m1** | **proliferation/cell cycle regulation** | p53 | ^17^ |
| **MET** | **Hs01565584_m1** | **proliferation/cell cycle regulation** | p53 | ^16^ |
| **MLH1** | **Hs00179866_m1** | **DNA damage and repair** | p53/AP1 | ^17^ |
| **MSH2** | **Hs00954125_m1** | **DNA damage and repair** | p53/AP1 | ^16^ |
| **MYC** | **Hs00905030_m1** | **proliferation/cell cycle regulation** | p53/NFBkB/p38 | ^17^,^65^,^44^ |
| **NLRP2** | **Hs01546932_m1** | **DNA damage and repair** | NFkB | ^66^ |
| **NOS2A** | **Hs01075523_m1** | **apoptosis** | NFkB | ^67^ |
| **NQO1** | **Hs00168547_m1** | **ROS** | NFkB | ^68^ |
| **OAS1** | **Hs00242943_m1** | **p38/JNK** | p38 | ^44^ |
| **P2RY13** | **Hs01090437_g1** | **UPR/ER Stress** | UPR target gene |  |
| **PARP1** | **Hs00911377_g1** | **necrosis/autophagy** |  |  |
| **PCNA** | **Hs00696862_m1** | **proliferation/cell cycle regulation** | p53/AP1 | ^17^ |
| **PGK1** | **Hs00943178_g1** | **housekeeping gene** |  |  |
| **PHLDA1** | **Hs00378285_g1** | **p38/JNK** | p38 | ^44^ |
| **PIDD** | **Hs01076855_g1** | **apoptosis** | p53 | ^69^ |
| **PLA2G6** | **Hs00895666_m1** | **UPR/ER Stress** | UPR target gene | ^70^ |
| **PMAIP1** | **Hs00560402_m1** | **apoptosis** | p53 | ^17^ |
| **POL2RB** | **Hs00265358_m1** | **housekeeping gene** |  |  |
| **PPP1R15A** | **Hs00921550_g1** | **UPR/ER Stress** | UPR target gene | ^71^ |
| **PRDX1** | **Hs03044568_g1** | **ROS** | NRF-2 upon oxidative stress | ^72^ |
| **PVR** | **Hs00197846_m1** | **necrosis/autophagy** | NRF-1 upon oxidative stress | ^73^ |
| **RAD51** | **Hs00947964_m1** | **DNA damage and repair** | transcriptionally regulated under stress (hypoxia) | ^48^ |
| **RB1** | **Hs01078075_m1** | **proliferation/cell cycle regulation** | p53 | ^16^ |
| **RIPK1** | **Hs00169407_m1** | **necrosis/autophagy** |  |  |
| **RPA1** | **Hs01556877_m1** | **proliferation/cell cycle regulation** |  |  |
| **RPLP0** | **Hs02992885_s1** | **housekeeping gene** |  |  |
| **RRM2B** | **Hs00968427_m1** | **DNA damage and repair** | p53 | ^74^ |
| **SENP2** | **Hs00989703_m1** | **apoptosis** | NFkB | ^75^ |
| **SESN1** | **Hs00902782_m1** | **DNA damage and repair** | p53 | ^17^ |
| **SFN** | **Hs00968567_s1** | **proliferation/cell cycle regulation** | p53 | ^76^ |
| **SHISA5** | **Hs00429977_m1** | **apoptosis** | p53 | ^77^ |
| **SIRT1** | **Hs01009000_m1** | **DNA damage and repair** | target of stress inducible miR34a and HuR | ^78^ |
| **SOD1** | **Hs00916176_m1** | **ROS** | NFkB | ^79^ |
| **SOD2** | **Hs04260076_g1** | **ROS** | NFkB | ^80^ |
| **SQSTM1** | **Hs01061917_g1** | **ROS** | NRF-2 upon oxidative stress | ^81^ |
| **SUMO1** | **Hs00830844_g1** | **DNA damage and repair** | target of stress inducible miR150 |  |
| **TAP73** | **Hs01056228_m1** | **proliferation/cell cycle regulation** | p53 | ^82^ |
| **TBP** | **Hs00427621_m1** | **housekeeping gene** |  |  |
| **TERT** | **Hs00972656_m1** | **proliferation/cell cycle regulation** | p53/NFBkB | ^83^ |
| **TNFRSF10A** | **Hs00269492_m1** | **apoptosis** | p53/NFBkB | ^84^ |
| **TNFRSF10B** | **Hs00366278_m1** | **apoptosis** | p53/NFBkB | ^17^ |
| **TNFRSF10C** | **Hs00182570_m1** | **apoptosis** | p53/NFBkB | ^85^ |
| **TNFRSF10D** | **Hs04187502_m1** | **apoptosis** | p53/NFBkB | ^17^ |
| **TP53** | **Hs99999147_m1** | **proliferation/cell cycle regulation** | p53/NFBkB | ^86^,^16^ |
| **TP53AIP1** | **Hs00986095_m1** | **apoptosis** | p53/NFBkB | ^87^ |
| **TP53I3** | **Hs00936520_m1** | **DNA damage and repair** | p53/NFBkB | ^17^ |
| **TP53INP1** | **Hs00264502_m1** | **DNA damage and repair** | p53/NFBkB | ^17^ |
| **TP63** | **Hs00978342_m1** | **proliferation/cell cycle regulation** | p53/NFBkB | ^16^ |
| **TP73** | **Hs01056230_m1** | **proliferation/cell cycle regulation** | p53/NFBkB | ^17^ |
| **TRAF1** | **Hs01090167_g1** | **apoptosis** | p53/NFBkB | ^88^ |
| **TRAF2** | **Hs00184192_m1** | **apoptosis** | p53/NFBkB | ^89^ |
| **TRPM3** | **Hs01558432_m1** | **p38/JNK** |  |  |
| **TXN** | **Hs00828652_m1** | **ROS** | NRF-2 upon oxidative stress | ^90^ |
| **XBP1** | **Hs00231936_m1** | **UPR/ER Stress** | UPR target gene | ^91^ |
| **XBP1(U)** | **Hs02856596_m1** | **UPR/ER Stress** | UPR target gene | ^91^ |
| **XIAP** | **Hs00745222_s1** | **apoptosis** | NFkB | ^92^ |
| **XPA** | **Hs00166045_m1** | **DNA damage and repair** | HIF1a | ^57^ |
| **XPC** | **Hs01104213_m1** | **DNA damage and repair** | p53/BRCA1/AP1 | ^17^ |
| **XRCC1** | **Hs00959834_m1** | **DNA damage and repair** | E2F1/AP1 | ^93^ |
| **XRCC3** | **Hs00193725_m1** | **DNA damage and repair** | transcr inducable by irradiation | ^94^ |
| **ZFP36** | **Hs00185658_m1** | **p38/JNK** | p38 | ^44^ |
| **ZMAT3** | **Hs00536976_m1** | **apoptosis** | p53 | ^95^ |

References:

1. Johnson, R. A. Transcriptional Repression by p53 through Direct Binding to a Novel DNA Element. *J. Biol. Chem.* **276,** 27716–27720 (2001).

2. Wu, M. *et al.* AMID is a p53-inducible gene downregulated in tumors. *Oncogene* **23,** 6815–6819 (2004).

3. Arakawa, T., Nakamura, M., Yoshimoto, T. & Yamamoto, S. The transcriptional regulation of human arachidonate 12-lipoxygenase gene by NF kappa B/Rel. *FEBS Lett.* **363,** 105–110 (1995).

4. Robles, A. I., Bemmels, N. A., Foraker, A. B. & Harris, C. C. APAF-1 is a transcriptional target of p53 in DNA damage-induced apoptosis. *Cancer Res.* **61,** 6660–6664 (2001).

5. Lv, G. *et al.* MicroRNA-451 regulates activating transcription factor 2 expression and inhibits liver cancer cell migration. *Oncol. Rep.* (2014). doi:10.3892/or.2014.3296

6. Zhang, C. *et al.* Transcriptional activation of the human stress-inducible transcriptional repressor ATF3 gene promoter by p53. *Biochem. Biophys. Res. Commun.* **297,** 1302–1310 (2002).

7. Kim, I., Xu, W. & Reed, J. C. Cell death and endoplasmic reticulum stress: disease relevance and therapeutic opportunities. *Nat. Rev. Drug Discov.* **7,** 1013–1030 (2008).

8. Adachi, Y. *et al.* ATF6 Is a Transcription Factor Specializing in the Regulation of Quality Control Proteins in the Endoplasmic Reticulum. *Cell Struct. Funct.* **33,** 75–89 (2008).

9. Verfaillie, T., Salazar, M., Velasco, G. & Agostinis, P. Linking ER Stress to Autophagy: Potential Implications for Cancer Therapy. *Int. J. Cell Biol.* **2010,** e930509 (2010).

10. Maskey, D. *et al.* ATG5 is induced by DNA-damaging agents and promotes mitotic catastrophe independent of autophagy. *Nat. Commun.* **4,** (2013).

11. Comincini, S. *et al.* microRNA-17 regulates the expression of ATG7 and modulates the autophagy process, improving the sensitivity to temozolomide and low-dose ionizing radiation treatments in human glioblastoma cells. *Cancer Biol. Ther.* **14,** 574–586 (2013).

12. Moiola, C. *et al.* Dynamic coregulatory complex containing BRCA1, E2F1 and CtIP controls ATM transcription. *Cell. Physiol. Biochem. Int. J. Exp. Cell. Physiol. Biochem. Pharmacol.* **30,** 596–608 (2012).

13. Wang, X. *et al.* HOXC9 directly regulates distinct sets of genes to coordinate diverse cellular processes during neuronal differentiation. *BMC Genomics* **14,** 830 (2013).

14. Graupner, V. *et al.* Differential regulation of the proapoptotic multidomain protein Bak by p53 and p73 at the promoter level. *Cell Death Differ.* **18,** 1130–1139 (2011).

15. Catz, S. D. & Johnson, J. L. Transcriptional regulation of bcl-2 by nuclear factor kappa B and its significance in prostate cancer. *Oncogene* **20,** 7342–7351 (2001).

16. Riley, T., Sontag, E., Chen, P. & Levine, A. Transcriptional control of human p53-regulated genes. *Nat. Rev. Mol. Cell Biol.* **9,** 402–412 (2008).

17. Wei, C.-L. *et al.* A global map of p53 transcription-factor binding sites in the human genome. *Cell* **124,** 207–219 (2006).

18. Grumont, R. J., Rourke, I. J. & Gerondakis, S. Rel-dependent induction of A1 transcription is required to protect B cells from antigen receptor ligation-induced apoptosis. *Genes Dev.* **13,** 400–411 (1999).

19. Lee, R. M., Gillet, G., Burnside, J., Thomas, S. J. & Neiman, P. Role of Nr13 in regulation of programmed cell death in the bursa of Fabricius. *Genes Dev.* **13,** 718–728 (1999).

20. Wang, Z., Zhang, B., Yang, L., Ding, J. & Ding, H.-F. Constitutive production of NF-kappaB2 p52 is not tumorigenic but predisposes mice to inflammatory autoimmune disease by repressing Bim expression. *J. Biol. Chem.* **283,** 10698–10706 (2008).

21. Miled, C., Pontoglio, M., Garbay, S., Yaniv, M. & Weitzman, J. B. A genomic map of p53 binding sites identifies novel p53 targets involved in an apoptotic network. *Cancer Res.* **65,** 5096–5104 (2005).

22. Copetti, T., Bertoli, C., Dalla, E., Demarchi, F. & Schneider, C. p65/RelA modulates BECN1 transcription and autophagy. *Mol. Cell. Biol.* **29,** 2594–2608 (2009).

23. Sax, J. K. *et al.* BID regulation by p53 contributes to chemosensitivity. *Nat. Cell Biol.* **4,** 842–849 (2002).

24. Chinnadurai, G., Vijayalingam, S. & Rashmi, R. BIK, the founding member of the BH3-only family proteins: mechanisms of cell death and role in cancer and pathogenic processes. *Oncogene* **27 Suppl 1,** S20–29 (2008).

25. Stehlik, C. *et al.* Nuclear factor (NF)-kappaB-regulated X-chromosome-linked iap gene expression protects endothelial cells from tumor necrosis factor alpha-induced apoptosis. *J. Exp. Med.* **188,** 211–216 (1998).

26. Slupianek, A. *et al.* BLM helicase is activated in BCR/ABL leukemia cells to modulate responses to cisplatin. *Oncogene* **24,** 3914–3922 (2005).

27. Baetz, D. *et al.* Nuclear factor-kappaB-mediated cell survival involves transcriptional silencing of the mitochondrial death gene BNIP3 in ventricular myocytes. *Circulation* **112,** 3777–3785 (2005).

28. Yakovlev, A. G. *et al.* BOK and NOXA are essential mediators of p53-dependent apoptosis. *J. Biol. Chem.* **279,** 28367–28374 (2004).

29. Thakur, S. *et al.* Regulation of BRCA1 Transcription by Specific Single-Stranded DNA Binding Factors. *Mol. Cell. Biol.* **23,** 3774–3787 (2003).

30. Rivera, A., Mavila, A., Bayless, K. J., Davis, G. E. & Maxwell, S. A. Cyclin A1 is a p53-induced gene that mediates apoptosis, G2/M arrest, and mitotic catastrophe in renal, ovarian, and lung carcinoma cells. *Cell. Mol. Life Sci. CMLS* **63,** 1425–1439 (2006).

31. Innocente, S. A., Abrahamson, J. L., Cogswell, J. P. & Lee, J. M. p53 regulates a G2 checkpoint through cyclin B1. *Proc. Natl. Acad. Sci. U. S. A.* **96,** 2147–2152 (1999).

32. Okamoto, K. & Beach, D. Cyclin G is a transcriptional target of the p53 tumor suppressor protein. *EMBO J.* **13,** 4816–4822 (1994).

33. Mori, T. *et al.* Cyclin K as a direct transcriptional target of the p53 tumor suppressor. *Neoplasia N. Y. N* **4,** 268–274 (2002).

34. St Clair, S. *et al.* DNA damage-induced downregulation of Cdc25C is mediated by p53 via two independent mechanisms: one involves direct binding to the cdc25C promoter. *Mol. Cell* **16,** 725–736 (2004).

35. Yun, J. *et al.* p53 negatively regulates cdc2 transcription via the CCAAT-binding NF-Y transcription factor. *J. Biol. Chem.* **274,** 29677–29682 (1999).

36. Hinata, K., Gervin, A. M., Jennifer Zhang, Y. & Khavari, P. A. Divergent gene regulation and growth effects by NF-kappa B in epithelial and mesenchymal cells of human skin. *Oncogene* **22,** 1955–1964 (2003).

37. Chu, I. M., Hengst, L. & Slingerland, J. M. The Cdk inhibitor p27 in human cancer: prognostic potential and relevance to anticancer therapy. *Nat. Rev. Cancer* **8,** 253–267 (2008).

38. Kreuz, S., Siegmund, D., Scheurich, P. & Wajant, H. NF-kappaB inducers upregulate cFLIP, a cycloheximide-sensitive inhibitor of death receptor signaling. *Mol. Cell. Biol.* **21,** 3964–3973 (2001).

39. Matsui, T. *et al.* Negative regulation of Chk2 expression by p53 is dependent on the CCAAT-binding transcription factor NF-Y. *J. Biol. Chem.* **279,** 25093–25100 (2004).

40. Samali, A., FitzGerald, U., Deegan, S. & Gupta, S. Methods for Monitoring Endoplasmic Reticulum Stress and the Unfolded Protein Response. *Int. J. Cell Biol.* **2010,** e830307 (2010).

41. Olivari, S., Galli, C., Alanen, H., Ruddock, L. & Molinari, M. A novel stress-induced EDEM variant regulating endoplasmic reticulum-associated glycoprotein degradation. *J. Biol. Chem.* **280,** 2424–2428 (2005).

42. Nishi, H. *et al.* Analysis of the epidermal growth factor receptor promoter: the effect of nuclear factor-kappaB. *Int. J. Mol. Med.* **11,** 49–55 (2003).

43. Zhou, A., Scoggin, S., Gaynor, R. B. & Williams, N. S. Identification of NF-kappa B-regulated genes induced by TNFalpha utilizing expression profiling and RNA interference. *Oncogene* **22,** 2054–2064 (2003).

44. Ferreiro, I. *et al.* Whole genome analysis of p38 SAPK-mediated gene expression upon stress. *BMC Genomics* **11,** 144 (2010).

45. Yan, Q.-W. *et al.* MZF1 possesses a repressively regulatory function in ERCC1 expression. *Biochem. Pharmacol.* **71,** 761–771 (2006).

46. Tanaka, K., Oda, N., Iwasaka, C., Abe, M. & Sato, Y. Induction of Ets-1 in endothelial cells during reendothelialization after denuding injury. *J. Cell. Physiol.* **176,** 235–244 (1998).

47. Li, N., Zhang, F., Li, S. & Zhou, S. Epigenetic silencing of MicroRNA-503 regulates FANCA expression in non-small cell lung cancer cell. *Biochem. Biophys. Res. Commun.* **444,** 611–616 (2014).

48. Scanlon, S. E. & Glazer, P. M. Hypoxic Stress Facilitates Acute Activation and Chronic Downregulation of Fanconi Anemia Proteins. *Mol. Cancer Res. MCR* (2014). doi:10.1158/1541-7786.MCR-13-0628

49. Tsai, Y.-S. *et al.* Areca nut induces miR-23a and inhibits repair of DNA double-strand breaks by targeting FANCG. *Toxicol. Sci. Off. J. Soc. Toxicol.* **123,** 480–490 (2011).

50. Chan, H., Bartos, D. P. & Owen-Schaub, L. B. Activation-dependent transcriptional regulation of the human Fas promoter requires NF-kappaB p50-p65 recruitment. *Mol. Cell. Biol.* **19,** 2098–2108 (1999).

51. Matsui, K., Fine, A., Zhu, B., Marshak-Rothstein, A. & Ju, S. T. Identification of two NF-kappa B sites in mouse CD95 ligand (Fas ligand) promoter: functional analysis in T cell hybridoma. *J. Immunol. Baltim. Md 1950* **161,** 3469–3473 (1998).

52. Christmann, M., Tomicic, M. T., Origer, J. & Kaina, B. Fen1 is induced p53 dependently and involved in the recovery from UV-light-induced replication inhibition. *Oncogene* **24,** 8304–8313 (2005).

53. Kwak, E. L., Larochelle, D. A., Beaumont, C., Torti, S. V. & Torti, F. M. Role for NF-kappa B in the regulation of ferritin H by tumor necrosis factor-alpha. *J. Biol. Chem.* **270,** 15285–15293 (1995).

54. Xia, C., Hu, J., Ketterer, B. & Taylor, J. B. The organization of the human GSTP1-1 gene promoter and its response to retinoic acid and cellular redox status. *Biochem. J.* **313 ( Pt 1),** 155–161 (1996).

55. Harrison, P. M. & Farzaneh, F. Regulation of HGF/SF gene expression in MRC-5 cells by N-acetylcysteine. *Biochem. Biophys. Res. Commun.* **279,** 108–115 (2000).

56. Bonello, S. *et al.* Reactive oxygen species activate the HIF-1alpha promoter via a functional NFkappaB site. *Arterioscler. Thromb. Vasc. Biol.* **27,** 755–761 (2007).

57. Liu, Y., Bernauer, A. M., Yingling, C. M. & Belinsky, S. A. HIF1α regulated expression of XPA contributes to cisplatin resistance in lung cancer. *Carcinogenesis* **33,** 1187–1192 (2012).

58. Lavrovsky, Y., Schwartzman, M. L., Levere, R. D., Kappas, A. & Abraham, N. G. Identification of binding sites for transcription factors NF-kappa B and AP-2 in the promoter region of the human heme oxygenase 1 gene. *Proc. Natl. Acad. Sci. U. S. A.* **91,** 5987–5991 (1994).

59. Imaizumi, K. *et al.* Molecular cloning of a novel polypeptide, DP5, induced during programmed neuronal death. *J. Biol. Chem.* **272,** 18842–18848 (1997).

60. Ammirante, M. *et al.* The activity of hsp90 alpha promoter is regulated by NF-kappa B transcription factors. *Oncogene* **27,** 1175–1178 (2008).

61. Wu, M. X., Ao, Z., Prasad, K. V., Wu, R. & Schlossman, S. F. IEX-1L, an apoptosis inhibitor involved in NF-kappaB-mediated cell survival. *Science* **281,** 998–1001 (1998).

62. Whitmarsh, A. J. A central role for p38 MAPK in the early transcriptional response to stress. *BMC Biol.* **8,** 47 (2010).

63. Tiwari, N. *et al.* Klf4 is a transcriptional regulator of genes critical for EMT, including Jnk1 (Mapk8). *PloS One* **8,** e57329 (2013).

64. Juin, P., Geneste, O., Gautier, F., Depil, S. & Campone, M. Decoding and unlocking the BCL-2 dependency of cancer cells. *Nat. Rev. Cancer* **13,** 455–465 (2013).

65. Duyao, M. P., Buckler, A. J. & Sonenshein, G. E. Interaction of an NF-kappa B-like factor with a site upstream of the c-myc promoter. *Proc. Natl. Acad. Sci. U. S. A.* **87,** 4727–4731 (1990).

66. Fontalba, A., Gutierrez, O. & Fernandez-Luna, J. L. NLRP2, an inhibitor of the NF-kappaB pathway, is transcriptionally activated by NF-kappaB and exhibits a nonfunctional allelic variant. *J. Immunol. Baltim. Md 1950* **179,** 8519–8524 (2007).

67. Geller, D. A. *et al.* Cytokine induction of interferon regulatory factor-1 in hepatocytes. *Surgery* **114,** 235–242 (1993).

68. Yao, K. S. & O’Dwyer, P. J. Involvement of NF-kappa B in the induction of NAD(P)H:quinone oxidoreductase (DT-diaphorase) by hypoxia, oltipraz and mitomycin C. *Biochem. Pharmacol.* **49,** 275–282 (1995).

69. Lin, Y., Ma, W. & Benchimol, S. Pidd, a new death-domain-containing protein, is induced by p53 and promotes apoptosis. *Nat. Genet.* **26,** 122–127 (2000).

70. Acosta-Alvear, D. *et al.* XBP1 controls diverse cell type- and condition-specific transcriptional regulatory networks. *Mol. Cell* **27,** 53–66 (2007).

71. Marciniak, S. J. *et al.* CHOP induces death by promoting protein synthesis and oxidation in the stressed endoplasmic reticulum. *Genes Dev.* **18,** 3066–3077 (2004).

72. Kim, Y.-J. *et al.* Human prx1 gene is a target of Nrf2 and is up-regulated by hypoxia/reoxygenation: implication to tumor biology. *Cancer Res.* **67,** 546–554 (2007).

73. Ardolino, M. *et al.* DNAM-1 ligand expression on Ag-stimulated T lymphocytes is mediated by ROS-dependent activation of DNA-damage response: relevance for NK-T cell interaction. *Blood* **117,** 4778–4786 (2011).

74. Nakano, K., Bálint, E., Ashcroft, M. & Vousden, K. H. A ribonucleotide reductase gene is a transcriptional target of p53 and p73. *Oncogene* **19,** 4283–4289 (2000).

75. Lee, M. H., Mabb, A. M., Gill, G. B., Yeh, E. T. H. & Miyamoto, S. NF-κB induction of the SUMO protease SENP2: A negative feedback loop to attenuate cell survival response to genotoxic stress. *Mol. Cell* **43,** 180–191 (2011).

76. Hermeking, H. *et al.* 14-3-3 sigma is a p53-regulated inhibitor of G2/M progression. *Mol. Cell* **1,** 3–11 (1997).

77. Bourdon, J.-C., Renzing, J., Robertson, P. L., Fernandes, K. N. & Lane, D. P. Scotin, a novel p53-inducible proapoptotic protein located in the ER and the nuclear membrane. *J. Cell Biol.* **158,** 235–246 (2002).

78. Milner, J. Cellular regulation of SIRT1. *Curr. Pharm. Des.* **15,** 39–44 (2009).

79. Rojo, A. I., Salinas, M., Martín, D., Perona, R. & Cuadrado, A. Regulation of Cu/Zn-superoxide dismutase expression via the phosphatidylinositol 3 kinase/Akt pathway and nuclear factor-kappaB. *J. Neurosci. Off. J. Soc. Neurosci.* **24,** 7324–7334 (2004).

80. Xu, Y. *et al.* Mutations in the promoter reveal a cause for the reduced expression of the human manganese superoxide dismutase gene in cancer cells. *Oncogene* **18,** 93–102 (1999).

81. Jain, A. *et al.* p62/SQSTM1 is a target gene for transcription factor NRF2 and creates a positive feedback loop by inducing antioxidant response element-driven gene transcription. *J. Biol. Chem.* **285,** 22576–22591 (2010).

82. Chen, X., Zheng, Y., Zhu, J., Jiang, J. & Wang, J. p73 is transcriptionally regulated by DNA damage, p53, and p73. *Oncogene* **20,** 769–774 (2001).

83. Yin, L., Hubbard, A. K. & Giardina, C. NF-kappa B regulates transcription of the mouse telomerase catalytic subunit. *J. Biol. Chem.* **275,** 36671–36675 (2000).

84. Liu, X., Yue, P., Khuri, F. R. & Sun, S.-Y. p53 upregulates death receptor 4 expression through an intronic p53 binding site. *Cancer Res.* **64,** 5078–5083 (2004).

85. Liu, X., Yue, P., Khuri, F. R. & Sun, S.-Y. Decoy receptor 2 (DcR2) is a p53 target gene and regulates chemosensitivity. *Cancer Res.* **65,** 9169–9175 (2005).

86. Wu, H. & Lozano, G. NF-kappa B activation of p53. A potential mechanism for suppressing cell growth in response to stress. *J. Biol. Chem.* **269,** 20067–20074 (1994).

87. O’Prey, J. *et al.* p53-mediated induction of Noxa and p53AIP1 requires NFkappaB. *Cell Cycle Georget. Tex* **9,** 947–952 (2010).

88. Schwenzer, R. *et al.* The human tumor necrosis factor (TNF) receptor-associated factor 1 gene (TRAF1) is up-regulated by cytokines of the TNF ligand family and modulates TNF-induced activation of NF-kappaB and c-Jun N-terminal kinase. *J. Biol. Chem.* **274,** 19368–19374 (1999).

89. Wang, C. Y., Mayo, M. W., Korneluk, R. G., Goeddel, D. V. & Baldwin, A. S. NF-kappaB antiapoptosis: induction of TRAF1 and TRAF2 and c-IAP1 and c-IAP2 to suppress caspase-8 activation. *Science* **281,** 1680–1683 (1998).

90. Hawkes, H.-J. K., Karlenius, T. C. & Tonissen, K. F. Regulation of the human thioredoxin gene promoter and its key substrates: a study of functional and putative regulatory elements. *Biochim. Biophys. Acta* **1840,** 303–314 (2014).

91. Byrd, A. E. & Brewer, J. W. Intricately Regulated: A Cellular Toolbox for Fine-Tuning XBP1 Expression and Activity. *Cells* **1,** 738–753 (2012).

92. Turner, D. J. *et al.* Bile salts induce resistance to apoptosis through NF-kappaB-mediated XIAP expression. *Ann. Surg.* **245,** 415–425 (2007).

93. Chen, D., Yu, Z., Zhu, Z. & Lopez, C. D. E2F1 regulates the base excision repair gene XRCC1 and promotes DNA repair. *J. Biol. Chem.* **283,** 15381–15389 (2008).

94. Yanagisawa, T., Urade, M., Yamamoto, Y. & Furuyama, J. Increased expression of human DNA repair genes, XRCC1, XRCC3 and RAD51, in radioresistant human KB carcinoma cell line N10. *Oral Oncol.* **34,** 524–528 (1998).

95. Hellborg, F. *et al.* Human wig-1, a p53 target gene that encodes a growth inhibitory zinc finger protein. *Oncogene* **20,** 5466–5474 (2001).
